# RUNX1 loss renders hematopoietic and leukemic cells dependent on IL-3 and sensitive to JAK inhibition

**DOI:** 10.1172/JCI167053

**Published:** 2023-10-02

**Authors:** Amy C. Fan, Yusuke Nakauchi, Lawrence Bai, Armon Azizi, Kevin A. Nuno, Feifei Zhao, Thomas Köhnke, Daiki Karigane, David Cruz-Hernandez, Andreas Reinisch, Purvesh Khatri, Ravindra Majeti

**Affiliations:** 1 Immunology Graduate Program,; 2 Institute for Stem Cell Biology and Regenerative Medicine,; 3 Cancer Institute,; 4 Department of Medicine, Division of Hematology, Stanford University School of Medicine, Stanford, California, USA.; 5 University of California Irvine School of Medicine, Irvine, California, USA.; 6 Cancer Biology Graduate Program, Stanford University School of Medicine, Stanford, California, USA.; 7 Medical Research Council (MRC) Molecular Haematology Unit and Oxford Centre for Haematology, University of Oxford, Oxford, United Kingdom.; 8 Division of Hematology, Medical University of Graz, Graz, Austria.; 9 Institute for Immunity, Transplantation and Infection, School of Medicine, and; 10 Center for Biomedical Informatics Research, Department of Medicine, Stanford University, Stanford, California, USA.

**Keywords:** Hematology, Inflammation, Hematopoietic stem cells, Human stem cells, Leukemias

## Abstract

Disease-initiating mutations in the transcription factor *RUNX1* occur as germline and somatic events that cause leukemias with particularly poor prognosis. However, the role of RUNX1 in leukemogenesis is not fully understood, and effective therapies for *RUNX1*-mutant leukemias remain elusive. Here, we used primary patient samples and a RUNX1-KO model in primary human hematopoietic cells to investigate how RUNX1 loss contributes to leukemic progression and to identify targetable vulnerabilities. Surprisingly, we found that RUNX1 loss decreased proliferative capacity and stem cell function. However, RUNX1-deficient cells selectively upregulated the IL-3 receptor. Exposure to IL-3, but not other JAK/STAT cytokines, rescued RUNX1-KO proliferative and competitive defects. Further, we demonstrated that RUNX1 loss repressed JAK/STAT signaling and rendered RUNX1-deficient cells sensitive to JAK inhibitors. Our study identifies a dependency of *RUNX1*-mutant leukemias on IL-3/JAK/STAT signaling, which may enable targeting of these aggressive blood cancers with existing agents.

## Graphical abstract

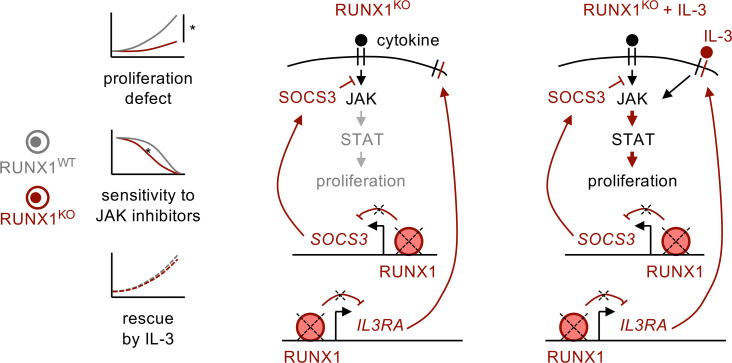

## Introduction

While aberrant immune signaling is a hallmark of many cancers, the mechanisms underlying dysregulated inflammatory signaling and the environmental cues driving disease pathogenesis are still being unraveled. In the last decade, the effect of different inflammatory stimuli in shaping the precancerous and malignant stages of leukemias has emerged as an area of great interest due to the clear contribution of inflammatory cytokines that can select for preleukemic and leukemic cells. Certain cytokines, such as IL-1, broadly promote leukemia survival (1), whereas others have mutation-specific effects, particularly on hematopoietic stem and progenitor cells (HSPCs) harboring preleukemic mutations. For example, IL-6 causes expansion of TET2-deficient HSPCs, whereas IFN-γ expands DNMT3A-mutant mouse HSPCs (2–4). With the advent of more-routine mutation screening and the development of targeted antiinflammatory therapeutics, determining the interplay between proinflammatory cytokines and mutations can identify potential therapeutic interventions.

RUNX1 is a master transcription factor commonly mutated in multiple cancers, with particularly high frequency in hematologic malignancies and breast, colon, and lung carcinomas. Specifically, somatic *RUNX1* mutations are most commonly found in myelodysplastic syndrome (MDS) and acute myeloid leukemia (AML) (5). Germline *RUNX1* mutations cause RUNX1 familial platelet disorder (RUNX1-FPD), which is characterized by thrombocytopenia, autoimmune diseases, and increased risk for MDS and AML, with an average age (in years) of onset in the mid-30s (6–8). *RUNX1* mutations in both germline and somatic myeloid malignancies are associated with resistance to chemotherapy and poor prognosis, with less than 20% overall survival at 2 years. In patients, *RUNX1*-mutant (*RUNX1*mut) leukemias are curable only by hematopoietic cell transplant. Thus, there is an urgent need for identifying therapies that can prevent leukemic progression in patients with germline *RUNX1* mutations and eradicate *RUNX1*mut AML in all patient populations.

Currently, it is unknown whether certain proinflammatory cytokines are necessary for mutant HSPC expansion or simply preferentially accelerate cell-intrinsic proliferative potential. *RUNX1*mut disease offers an interesting model for 2 reasons. First, mutations in *RUNX1* are by definition preleukemic in individuals with RUNX1-FPD, who have increased prevalence of autoimmune disease and likely a proinflammatory milieu. Second, *RUNX1*mut preleukemic clones are not detected in clonal hematopoiesis — the aberrant expansion of blood cell clones — indicating that they are not inherently more proliferative, unlike other preleukemic mutations commonly found in clonal hematopoiesis, such as *DNMT3A* and *TET2* (9–11). As there is accumulating evidence that RUNX1 interacts with multiple inflammatory signaling pathways (12–14), we sought to investigate whether *RUNX1*mut cells are dependent on specific inflammatory stimuli for expansion.

Here, using CRISPR/Cas9 and AAV6–medicated homology-directed repair (HDR), we established a model of RUNX1 loss in human HSPCs that recapitulates known phenotypes. We found that RUNX1 loss reduced stem cell activity and proliferation in vitro and in vivo in cytokine-poor environments. RUNX1 KO also downregulated JAK/STAT signaling through upregulation of SOCS3 expression. We show that exogenous IL-3 could mitigate the RUNX1-KO proliferative and competitive defects, which were mediated through upregulation of IL-3 receptor in RUNX1-deficient cells. Finally, we show that this dependence on IL-3/JAK/STAT signaling allowed for targeting of RUNX1-deficient cells using JAK inhibitors in both RUNX1-KO HSPCs and primary AML patient samples.

## Results

### RUNX1 loss in human CD34^+^ HSPCs by CRISPR/AAV6-mediated knock-in of fluorescent reporters.

**Figure 1:**
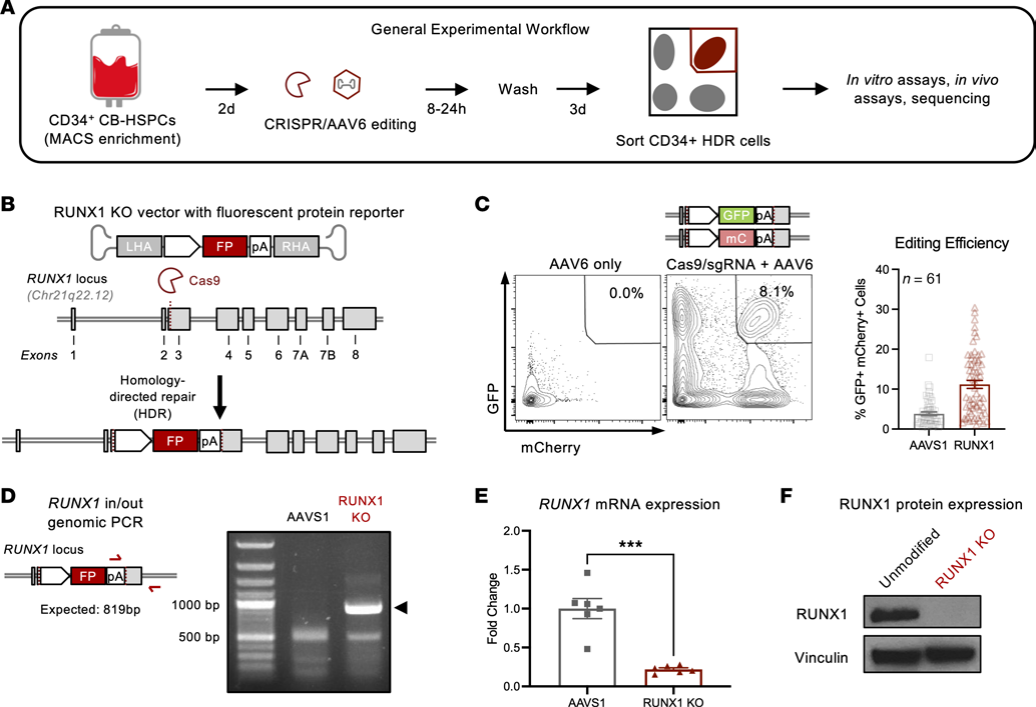
Targeting the endogenous *RUNX1* locus in human HSPCs using CRISPR and homology directed repair. (**A**) Umbilical CB is enriched for CD34 using magnetic activated cell sorting (MACS) and expanded in serum-free media with SCF, TPO, FLT3L, IL-6, and UM-171. After 2 days, cells are nucleofected with *RUNX1*- or *AAVS1*- targeting sgRNA-Cas9 ribonucleoprotein and exposed to AAV6 carrying donor DNA for 8 hours. Three days after editing, CD34^+^mCherry^+^GFP^+^ cells are sorted and plated for in vitro and in vivo assays or molecular profiling. (**B**) Recombinant AAV6 vector carries arms of homology flanking fluorescent reporter (FP) transgenes encoding GFP or mCherry as donor DNA for HDR at DNA double-stranded breaks generated by *RUNX1*-targeting sgRNA-Cas9. LHA, left homology arm; RHA, right homology arm; pA, poly-A. (**C**) Left: Biallelic modified GFP^+^mCherry^+^ double-positive cells are not present in controls lacking sgRNA-Cas9. mC, mCherry. Right: Quantification of double-positive HDR editing efficiency at the *AAVS1* safe harbor locus and *RUNX1* locus in CD34^+^ HSPCs. *n* = 61 CB donors. (**D**) Left: Schematic of in/out PCR spanning target-donor junction to confirm integration of the RUNX1-KO vector in the endogenous RUNX1 locus. Right: Agarose gel showing in/out PCR product in RUNX1-KO CD34^+^ HSPCs. (**E**) qPCR detection of fold change in RUNX1 expression relative to AAVS1 control and normalized to HPRT1. Unpaired *t* test: *** *P* < 0.001. *n* = 2 CB donors, 3 replicates. (**F**) Western blot of RUNX1 protein in unmodified and RUNX1-KO CD34^+^ HSPCs.

Because both germline and somatic *RUNX1* mutations cause hypomorphic, loss-of-function, or dominant-negative phenotypes (15, 16), we used CRISPR/Cas9 and AAV6-HDR to knock out RUNX1 in primary human CD34^+^ HSPCs (Figure 1A). Specifically, we introduced AAV6 vectors carrying donor DNA with fluorescent protein reporters flanked by homology arms to the region upstream and downstream of an exon 3 cut site introduced by a *RUNX1*-targeting sgRNA complexed to the Cas9 protein (Figure 1B). Fluorescent protein expression enabled us to isolate HSPCs with confirmed HDR by FACS for further evaluation (Figure 1C).

To evaluate the effect of complete RUNX1 loss on hematopoiesis —which occurs in RUNX1-FPD, where loss of the second *RUNX1* allele is the most common secondary mutation (17, 18) — we simultaneously introduced 2 fluorescent reporters and isolated GFP^+^mCherry^+^ cells with biallelic disruption of the *RUNX1* loci (Figure 1C). We introduced fluorescent reporters into the *AAVS1* safe harbor locus using a previously reported vector as a control (19) (Figure 1C). Biallelic HDR efficiency was variable among donors, with an average of 3.81% for the *AAVS1* locus and 11.2% for the *RUNX1* locus in cord blood (CB) HSPCs (Figure 1C), and an average of 9.58% for the *AAVS1* locus and 5.26% for the *RUNX1* locus in adult mobilized HSPCs (Supplemental Figure 1A; supplemental material available online with this article; https://doi.org/10.1172/JCI167053DS1). We confirmed that *RUNX1*-targeting AAV6 donor DNA integrated into the *RUNX1* locus, and that GFP^+^mCherry^+^ HSPCs had decreased *RUNX1* mRNA and protein levels compared with controls (Figure 1, D–F).

### RUNX1 KO causes hematopoietic differentiation and stem cell defects.

**Figure 2:**
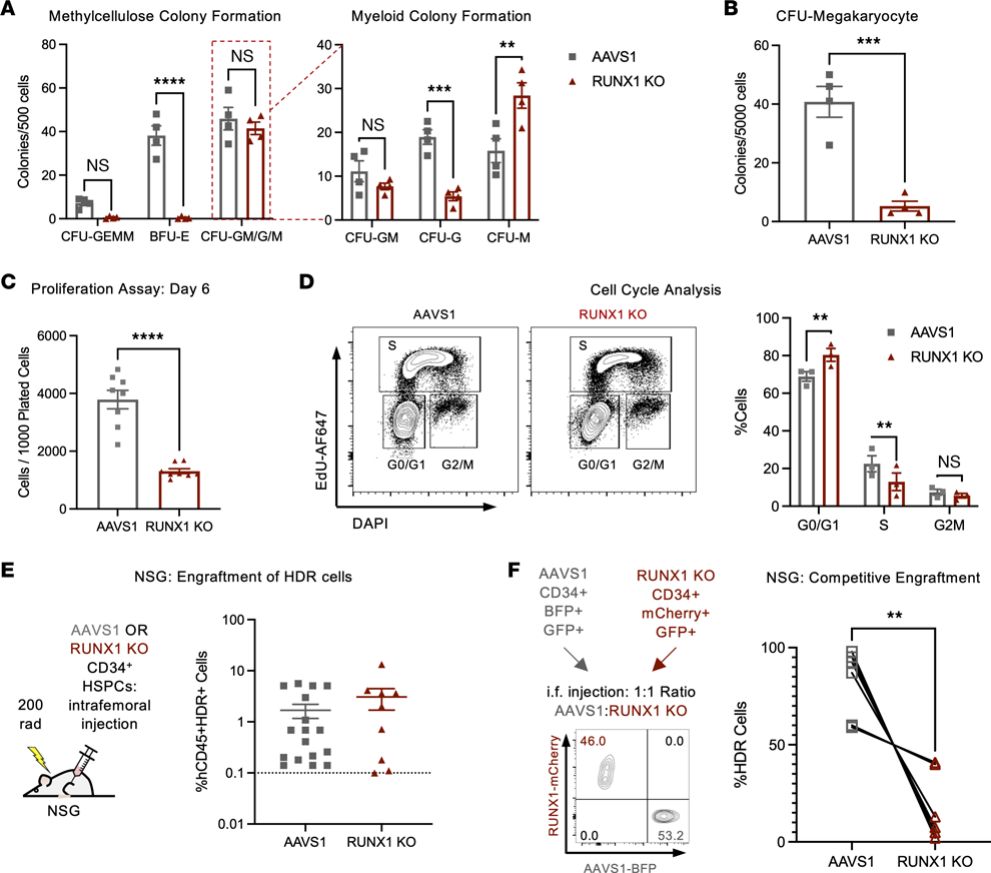
*RUNX1* loss in human HSPCs causes hematopoietic and stem cell defects. (**A**) CD34^+^ HDR HSPCs were plated in methylcellulose-based colony-forming assays and assessed for colony formation at 14 days. *n* = 4 CB. Two-Way ANOVA, Šidák’s multiple-comparison test: **P < 0.01, ***P < 0.001, ****P < 0.0001. (**B**) CD34^+^ HDR HSPCs were plated in collagen-based megakaryocyte colony-forming assays, and colonies were quantified after 10 days. *n* = 4 CB. Paired *t* test: ***P < 0.001. (**C**) CD34^+^ HDR HSPCs were plated in stem retention media (serum-free media with SCF, TPO, and FLT3L) and analyzed by flow cytometry for cell count at days 6. *n* = 8 CB donors. Paired *t* test: ****P < 0.0001. BFU, burst-forming units. GEMM, granulocyte, erythrocyte, monocyte, megakaryocyte. (**D**) CD34^+^ HDR HSPCs were incubated with EdU for 2 hours, stained with DAPI, and evaluated for cell-cycle status. *n* = 3 CB. Two-way ANOVA, Šidák’s multiple-comparison test: **P < 0.01. (**E**) CD34^+^ HDR HSPCs were injected intrafemorally into sublethally irradiated NSG mice, and human CD45^+^ HDR^+^ engraftment was evaluated upon sacrifice (at 24–26 weeks after transplantation). Unpaired *t* test NS (not significant). *n* = 5 CB donors, 9–18 mice. (**F**) BFP^+^ AAVS1 and mCherry^+^ RUNX1-KO cells were injected intrafemorally at a 1:1 ratio into NSG mice, and relative engraftment within the human CD45^+^ compartment was evaluated 18 weeks after transplantation. The FACS plot indicates representative ratio upon injection. The graph shows relative engraftment at 18 weeks; paired *t* test: **P < 0.01. *n* = 3 CB donors, 6 mice.

To evaluate the effect of RUNX1 loss on hematopoietic differentiation in vitro, we cultured control and RUNX1-KO CD34^+^ HSPCs in media with defined cytokines supporting megakaryocytic, erythroid, or myeloid differentiation. Consistent with RUNX1-FPD patient characteristics and cytopenias commonly associated with MDS, RUNX1-KO cells showed defective megakaryocytic and erythroid differentiation. RUNX1 KO decreased maturation into CD41^++^CD61^++^ mature megakaryocytes and CD71^+^glycophorin A^+^ (CD71^+^GPA^+^) erythroblasts, and ablated megakaryocytic and erythroid colony formation (Figure 2, A and B, and Supplemental Figure 1, B–D). Further, while total myeloid colony-forming potential was unaffected, RUNX1-KO cells preferentially differentiated into monocytes compared with granulocytes in both liquid-differentiation and colony-forming assays (Figure 2A and Supplemental Figure 1, B and E).

Next, we sought to determine how RUNX1 loss affects proliferation and self-renewal. We cultured control and RUNX1-KO CD34^+^ HSPCs in stem retention media: serum-free minimal media supplemented with stem cell factor (SCF), thrombopoietin (TPO), and Fms-related tyrosine kinase 3 ligand (FLT3L). RUNX1 KO reduced total cell number (Figure 2C and Supplemental Figure 1F), which we reasoned could be due to either decreased cell cycling or increased cell death. Staining for EdU/DAPI and DAPI/annexin V showed that RUNX1 KO caused a G_1_-to-S block but did not affect cell death, indicating that RUNX1 loss caused a proliferative defect (Figure 2D and Supplemental Figure 1G).

To evaluate the self-renewal capacity of RUNX1-KO HSPCs, we intrafemorally transplanted control or RUNX1-KO HSPCs into sublethally irradiated immunodeficient NSG mice. RUNX1-KO cells transplanted alone engrafted at levels comparable to those of AAVS1 controls (Figure 2E and Supplemental Figure 1H). However, when RUNX1-KO cells were cotransplanted with AAVS1 control HSPCs, human cell engraftment was biased toward controls, revealing a RUNX1-KO competitive defect (Figure 2F and Supplemental Figure 1I). Together, these differentiation defects recapitulated key characteristics of the preleukemic state found in RUNX1-FPD and MDS patients, namely thrombocytopenia, anemia, and neutropenia. While the stem-cell capacity of RUNX1-FPD patients has yet to be evaluated, MDS HSPCs exhibit similarly poor ex vivo growth and poor engraftment in immunodeficient mice (20).

### Increased NF-κB in RUNX1-KO HSPCs contributes to monocytic differentiation but not resistance to inflammatory stress.

To characterize the molecular mechanisms contributing to these differentiation and stem- cell phenotypes, we performed RNA-Seq and an assay for transposase-accessible chromatin with high-throughput sequencing (ATAC-Seq) on RUNX1-KO HSPCs. RUNX1 KO primarily resulted in the downregulation of gene transcription (576 downregulated genes, 365 upregulated genes) and redistribution of chromatin accessibility involved in gene regulation, particularly the closing of distal intergenic regions and opening of gene promoters (Supplemental Figure 2, A and B). Transcription factor motif and footprinting analysis confirmed that RUNX motifs were less accessible, and gene set enrichment analysis (GSEA) of RNA-Seq data showed that RUNX target genes were downregulated in RUNX1-KO cells (Supplemental Figure 2, C–E).

Consistent with the severe erythroid and megakaryocyte differentiation defects, gene expression, chromatin accessibility, and transcription factor activity associated with erythro-megakaryocytic differentiation were downregulated according to both RNA-Seq and ATAC-Seq (Supplemental Figure 2, A, D, and E and Supplemental Figure 3). The master transcription factor *GATA1* and many genes involved in erythroid (e.g., *GYPA*, *HBB*, *KLF1*) and megakaryocyte differentiation (e.g., *GP9*, *ITGA2B*, *ITGB3*, *PF4*, *VWF*) were downregulated and/or less accessible in RUNX1-KO cells (Supplemental Figure 2A and Supplemental Figure 3A). GATA family members and the megakaryocyte transcription factor SRF also showed decreased activity, as evidenced by decreases in transcription factor motif accessibility and in expression of transcription factor target genes (Supplemental Figure 2, D and E). Interestingly, while most gene programs with differential expression, including erythroid (“heme metabolism”) gene sets, were regulated by RUNX1 at gene promoters, megakaryocyte (“coagulation”) gene sets were instead enriched at differentially accessible enhancers (Supplemental Figure 3, B and C).

We next evaluated which myeloid gene programs and transcription factors were upregulated and therefore likely contributing to the myeloid-skewed differentiation phenotype seen in RUNX1-KO cells. RUNX1 KO increased expression of NF-κB gene programs and target genes, and NF-κB motifs were significantly enriched across the genome and particularly in gene-regulatory regions (Supplemental Figure 2, D–F, and Supplemental Figure 3B). To investigate the contribution of increased NF-κB signaling to RUNX1-KO HSPC myeloid differentiation, we plated RUNX1-KO HSPCs in liquid differentiation assays in the presence or absence of inhibitors targeting NF-κB or IκKβ. We found that inhibition of the NF-κB pathway in RUNX1-KO HSPCs reduced CD14^+^ monocyte differentiation, indicating a role for this pathway in RUNX1-KO myeloid differentiation (Supplemental Figure 4A).

Finally, previous studies have found that increased NF-κB activity can protect HSPCs from additional stress induced by external inflammatory pressure (21, 22). This has been proposed to be one mechanism by which the increased inflammatory signaling in MDS HSPCs, which exhibit stem cell-proliferative and competitive defects, can be selected by inflammation to outcompete WT HSPCs (12, 16). RUNX1 KO upregulated multiple inflammatory signaling pathways in HSPCs (Supplemental Figure 2F and Supplemental Figure 3B), raising the possibility that additional inflammatory stress may select for RUNX1-KO HSPCs. Because increased NF-κB in mouse HSPCs protects against TNF-α–induced apoptosis (21), we first cultured RUNX1-KO HSPCs in stem retention media with or without TNF-α and measured cell number and viability. Surprisingly, exposure to TNF-α killed both AAVS1 control and RUNX1-KO HSPCs, and RUNX1 loss, rather than promoting, reduced cell viability in the presence of TNF-α (Supplemental Figure 4B). Exposure to other inflammatory stressors that have been reported to promote clonal outgrowth of hematopoietic cells also did not result in preferential expansion of RUNX1-KO cells (Supplemental Figure 4C). Thus, increased NF-κB activity contributed to the myeloid skew in RUNX1-KO HSPCs but did not confer resistance to inflammatory stress.

### Repressed JAK/STAT signaling decreases proliferation of RUNX1-KO cells.

**Figure 3:**
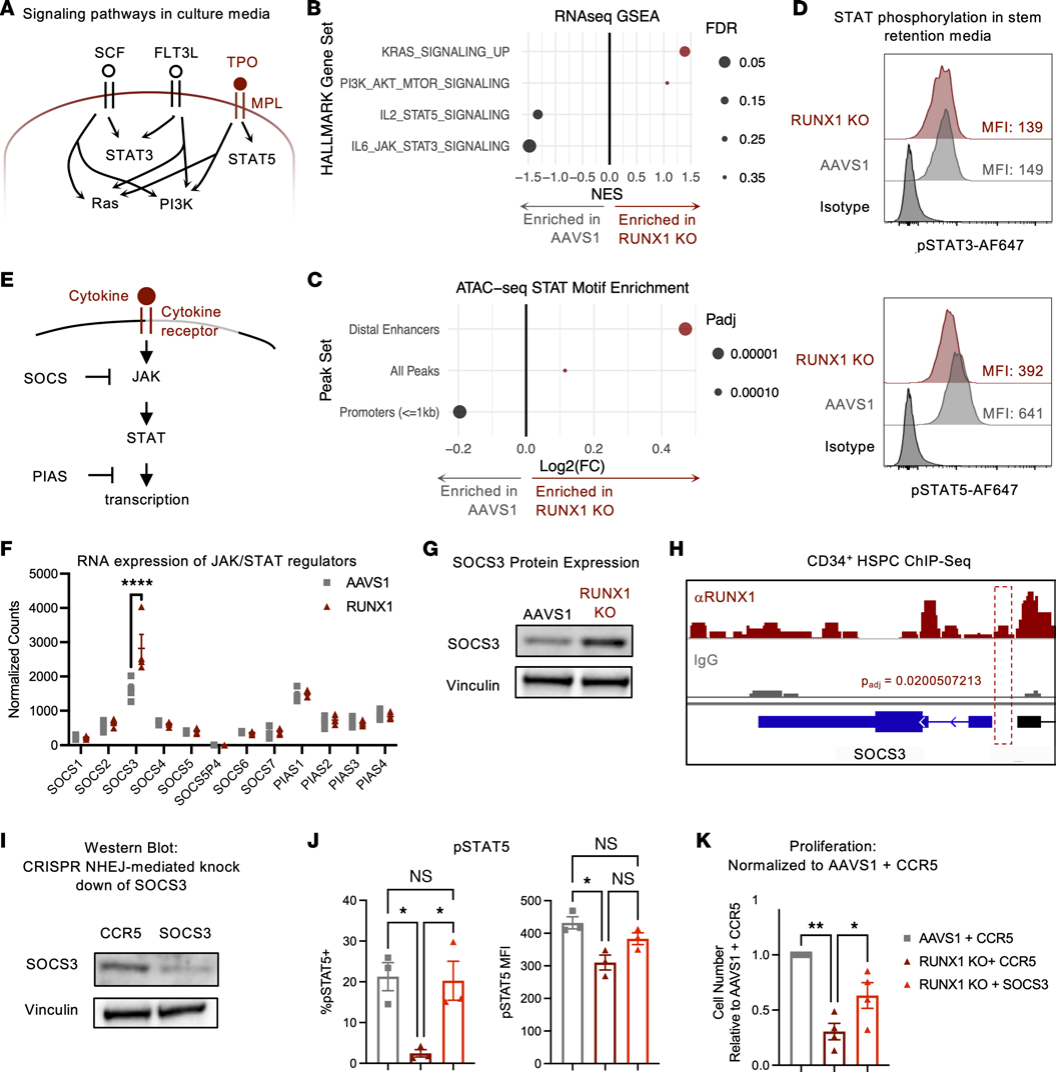
RUNX1 KO reduces JAK/STAT signaling. (**A**) Schematic of signaling pathways stimulated by stem retention media used for proliferation assay downstream of SCF, FLT3L, and TPO. (**B**) RNA-Seq HALLMARK GSEA of signaling gene sets in CD34^+^ HSPCs. NES, normalized enrichment score. (**C**) Change in ATAC-Seq peak transcription factor motif enrichment in RUNX1-KO relative to AAVS1 control cells in all differentially accessible ATAC-Seq peaks (“All Peaks”), promoters (≤1 kb), and distal enhancers. (**D**) Representative intracellular staining of pSTAT3 and pSTAT5 in CD34^+^ HDR HSPCs cultured in stem-retention media for *n* = 2 and *n* = 9 CB donors, respectively. (**E**) Schematic of cytokine receptor signaling through JAK/STAT suppressed by SOCS and PIAS proteins. (**F**) Gene expression of *SOCS* and *PIAS* JAK/STAT signaling negative feedback regulators on day 10 after editing. *n* = 4 CB donors. DESeq2 Wald test and Benjamini-Hochberg multiple test correction, ****q < 0.0001. (**G**) Western blot of SOCS3 protein in AAVS1 control and RUNX1-KO CD34^+^ HSPCs. (**H**) Genome tracks of RUNX1 and IgG control ChIP-Seq of mobilized CD34^+^ HSPCs at the *SOCS3* locus. (**I**) Western blot for SOCS3 in cells with *CCR5*-targeting sgRNA or *SOCS3*-targeting sgRNAs. (**J**) CD34^+^ HDR HSPCs were plated in stem retention media for 6 days and analyzed by flow cytometry for pSTAT5. pSTAT5 positivity was gated based on isotype controls. *n* = 3 CB donors. One-way ANOVA, Dunnett’s multiple-comparison test: *P < 0.05. (**K**) CD34^+^ HDR HSPCs were plated in stem retention media for 6 days and analyzed by flow cytometry for cell count using CountBright beads. Cell counts were normalized to AAVS1 + CCR5 control. *n* = 4 CB donors. One-way ANOVA, Dunnett’s multiple-comparison test: *P < 0.05, **P < 0.01.

To identify other mechanisms that select for RUNX1-deficient cells, we first evaluated whether defects in cytokine signaling may contribute to the RUNX1-KO HSPC proliferative defect. Of the major signaling pathways supporting HSPC survival and growth in stem retention media conditions, only JAK/STAT transcriptional activity was downregulated (Figure 3, A and B). Additionally, STAT motifs were depleted at accessible gene promoters (Figure 3C). Because cytokines supporting HSPC growth activate STAT3 and STAT5 (23) (Figure 3A), we evaluated phosphorylation of STAT3 (pSTAT3) and pSTAT5 in RUNX1-KO HSPCs and found that pSTAT5, but not pSTAT3, was decreased (Figure 3D).

As the only cytokine in our culture media that signals through STAT5 is TPO, it is possible that this decreased JAK/STAT signaling was due to deficient signaling of TPO through its receptor MPL (Figure 3A). However, augmenting TPO levels in cell culture did not increase RUNX1-KO or AAVS1 control cell number, indicating that TPO was present at saturating levels in stem retention media (Supplemental Figure 5A). Additionally, MPL expression remained unchanged in RUNX1-KO cells (Supplemental Figure 5B). Therefore, RUNX1 loss likely regulated JAK/STAT signaling downstream of the TPO receptor.

Activity of JAK and STAT is negatively regulated by SOCS and PIAS family proteins, respectively (24) (Figure 3E). In our study of these factors, RUNX1 KO upregulated expression of only *SOCS3* in HSPCs, and analysis of ChIP-Seq data showed that RUNX1 bound to the *SOCS3* promoter (Figure 3, F–H). Thus, RUNX1 likely directly repressed *SOCS3* expression, and loss of RUNX1 upregulated SOCS3, inhibiting JAK/STAT signaling. To determine whether knocking down SOCS3 can augment STAT5 activation and thereby increase cell growth in RUNX1-KO cells, we used CRISPR/Cas9-mediated nonhomologous end joining insertion/deletion mutations (NHEJ-indels) to target the *SOCS3* locus or the *CCR5* locus, which has previously been demonstrated to be a safe harbor locus (25). SOCS3 knockdown rescued STAT5 phosphorylation and partially rescued the proliferative defects of RUNX1-KO cells (Figure 3, I–K). Additionally, introducing shRNAs targeting *SOCS3* into the *RUNX1*-targeting AAV6 vectors resulted in similar increases in STAT5 phosphorylation and proliferation (Supplemental Figure 5, C–F). Together, these results demonstrate that RUNX1 loss repressed JAK signaling, which in turn repressed cell growth.

### IL-3 rescues RUNX1 KO proliferative and competitive defects in vitro and in vivo.

Many inflammatory cytokines signal through JAK/STAT and have been shown to expand mutant HSPCs. Thus, we investigated whether JAK/STAT cytokines can reverse the RUNX1-KO proliferative and competitive defects. Specifically, because we observed decreased pSTAT5, we first focused on members of the common β chain family of cytokines that also signal through STAT5, with established roles in promoting hematopoietic growth: GM-CSF, IL-3, and IL-5 (26). Additionally, because *RUNX1* mutations have been reported to increase colony formation in response to granulocyte colony-stimulating factor (G-CSF) (12), we also assayed the effect of G-CSF on RUNX1-KO cell growth.

**Figure 4:**
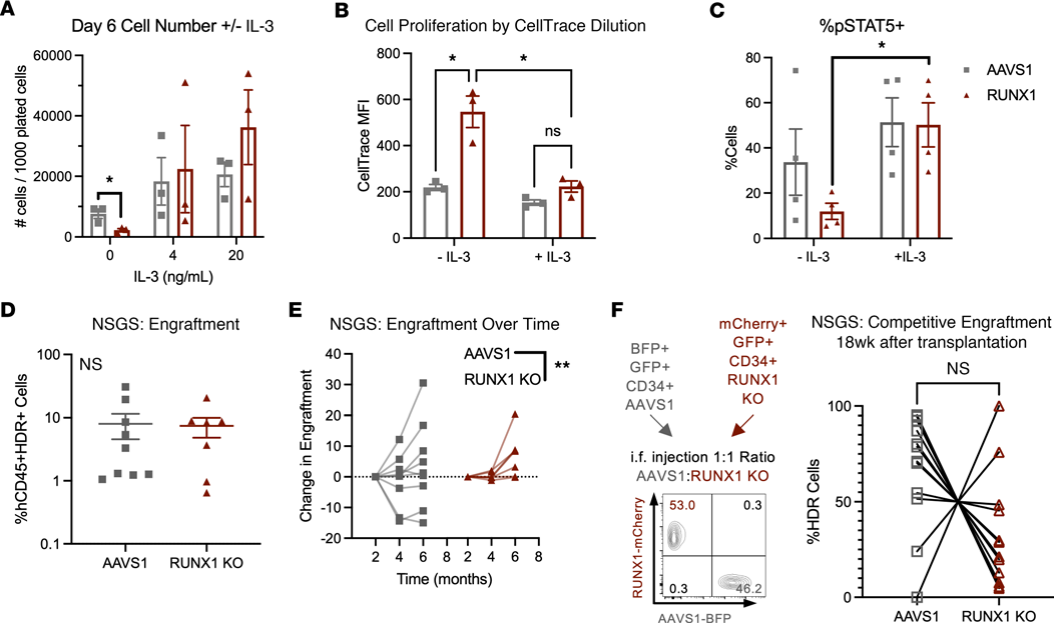
RUNX1 KO sensitizes to IL-3 rescue of proliferation and competitive defects. (**A**) CD34^+^ HDR HSPCs were plated in stem retention media and supplemented with 4 or 20 ng/mL IL-3. Cell count was determined at 6 days by flow cytometry using CountBright beads. *n* = 3 CB. Two-way ANOVA, Šidák’s multiple-comparison test: *P < 0.05. (**B**) CD34^+^ HDR HSPCs were labeled with CellTrace Violet and plated in stem retention media with or without 10 ng/mL IL-3. CellTrace MFI was determined after 4 days. *n* = 3 CB. Two-way ANOVA, Šidák’s multiple-comparison test: *P < 0.05. (**C**) pSTAT5^+^ cells were gated based on isotype controls and quantified in CD34^+^ HDR HSPCs plated in stem retention media with or without 10 ng/mL IL-3 after 7 days. *n* = 4 CB. Two-way ANOVA, Šidák’s multiple-comparison test: *P < 0.05. (**D**) CD34^+^ HDR HSPCs were injected intrafemorally into sublethally irradiated NSGS mice, and hCD45^+^HDR^+^ engraftment was evaluated upon sacrifice (at 24–26 weeks after transplantation). *n* = 4 CB, 7–8 mice. Unpaired *t* test. (**E**) CD34^+^ HDR HSPCs were injected intrafemorally into sublethally irradiated NSGS mice and hCD45^+^HDR^+^ engraftment monitored over time using bone marrow aspirates (at 8–10 weeks or 16–18 weeks after transplantation) and upon sacrifice (at 24–26 weeks after transplantation). *n* = 3 CB, 16 mice. Two-way ANOVA: *P < 0.05. (**F**) AAVS1 and RUNX1-KO cells were injected in a 1:1 ratio intrafemorally into sublethally irradiated NSGS mice and relative engraftment at 18 weeks was ascertained using bone marrow aspirates. *n* = 3 CB, 13 mice. Paired *t* test.

**Figure 5:**
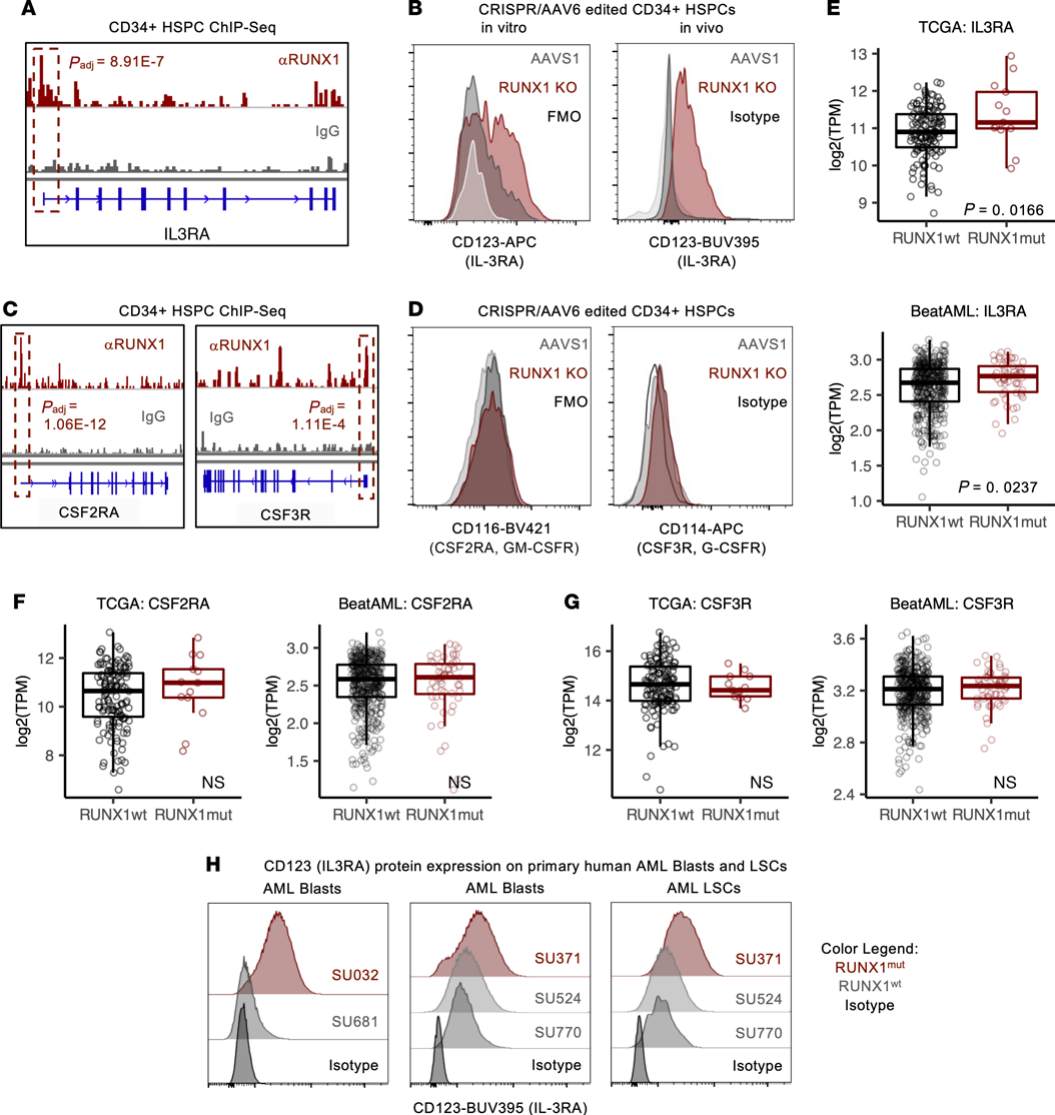
RUNX1 loss upregulates IL-3 receptor in HSPCs and leukemia. (**A**) Genome tracks of RUNX1 and IgG control ChIP-Seq of mobilized CD34^+^ HSPCs at the *IL3RA* locus. (**B**) Representative IL-3RA protein expression on CD34^+^ HDR HSPCs after 6 days of culture (left, in vitro) and from hCD45^+^HDR^+^ cells isolated from transplanted NSGS mice (right, in vivo); *n* = 3 CB each. (**C**) Genome tracks of RUNX1 and IgG control ChIP-Seq of mobilized CD34^+^ HSPCs at *CSF2RA* and *CSF3R* loci. (**D**) Representative CD116 (GM-CSFR) and CD114 (G-CSFR) protein expression on CD34^+^ HDR HSPCs after 6 days of culture; *n* = 3 CB each. (**E**) *IL3RA*, (**F**) *CSF2RA*, and (**G**) *CSF3R* transcript levels in RUNX1wt and RUNX1mut AML from TCGA and BeatAML. Unpaired *t* test. (**F**) IL-3RA protein expression on mutation profile–matched RUNX1mut and RUNX1wt Lin^–^CD45^lo^ blasts and CD34^+^CD38^–^TIM3^+^CD99^+^ LSCs in primary human AML samples.

We cultured control and RUNX1-KO HSPCs in stem retention media supplemented with G-CSF, GM-CSF, IL-3, or IL-5. While G-CSF increased RUNX1-KO cell number, RUNX1-KO cells were still decreased in comparison to AAVS1 controls (Supplemental Figure 5G). Notably, exposure to IL-3, but not GM-CSF or IL-5, rescued the RUNX1-KO in vitro growth defect, increasing RUNX1-KO cell numbers to levels comparable to or even greater than those for AAVS1 controls (Figure 4A; extended data, Figure 5H). IL-3 augmented cell numbers by inducing RUNX1-KO cell proliferation, as measured by CellTrace dilution, and rescued STAT5 phosphorylation (Figure 4, B and C, and Supplemental Figure 5I).

To determine whether IL-3 also augments RUNX1-KO cell growth in vivo, we transplanted RUNX1-KO HSPCs into NSG mice expressing ectopic human SCF, GM-CSF, and IL-3 (NSGS mice; ref. 27). Importantly, mouse and human IL-3 are not cross-reactive (28), and NSGS serum concentrations of human IL-3 are similar to the concentrations that rescued cell growth in our in vitro assays. While no differences in engraftment were observed between AAVS1 control and RUNX1-KO HSPCs in NSGS mice (Figure 4D and Supplemental Figure 5J), RUNX1-KO cells expanded to a greater degree over time (Figure 4E and Supplemental Figure 5K). Moreover, cotransplantation of RUNX1-KO and AAVS1 control HSPCs in NSGS mice also mitigated the competitive disadvantage exhibited by RUNX1-KO cells in NSG mice (Figure 4F and Supplemental Figure 5L). Thus, IL-3 could rescue the proliferative and competitive defects observed in RUNX1-KO HSPCs.

### RUNX1 loss in HSPCs and leukemic cells increases expression of IL-3RA.

We hypothesized that the selective hypersensitivity of RUNX1-KO HSPCs to IL-3 may be mediated by RUNX1 regulation of the IL-3–specific subunit of the IL-3 receptor: IL-3RA. Indeed, RUNX1 bound the *IL3RA* locus, and RUNX1-KO HSPCs expressed higher levels of IL-3RA compared with AAVS1 controls in both in vitro culture and xenotransplanted mice (Figure 5, A and B). In contrast, although RUNX1 bound the *CSF2RA* and *CSF3R* loci, encoding the GM-CSF and G-CSF receptors, respectively, protein levels of both receptors were unchanged in RUNX1-KO HSPCs (Figure 5, C and D).

Because *RUNX1* is commonly mutated in AML, it is possible that RUNX1 loss contributes to leukemia pathogenesis by increasing IL-3RA expression. Consistent with our RUNX1-KO HSPC model, *RUNX1*mut AML cells expressed higher levels of *IL3RA* transcripts compared with *RUNX1*-WT (*RUNX1*wt) AML cells, with no difference in *CSF2RA* or *CSF3R* expression (Figure 5, E–G). To ascertain that this upregulation was not due to mutations that may commonly co-occur with *RUNX1*, we identified *RUNX1*mut and *RUNX1*wt normal-karyotype patient samples with similar mutation profiles (Supplemental Table 1). *RUNX1*mut AML blasts (SU032 and SU371) expressed higher levels of IL-3RA than matched *RUNX1*wt AML blasts (SU681 and SU524/SU770, respectively) (Figure 5H).

IL-3RA has also been described as a marker for leukemia stem cells, which are associated with chemoresistance and disease relapse (29, 30). In the CD34^+^CD38^–^TIM3^+^CD99^+^ leukemia stem cell–enriched (LSC-enriched) fraction, *RUNX1*mut LSCs (SU371) exhibited higher expression of IL-3RA compared with matched *RUNX1*wt LSCs (SU524, SU770) (Figure 5H). Therefore, RUNX1 loss upregulated IL-3RA in primary human hematopoietic and leukemic cells.

### RUNX1 loss in HSPCs and leukemic cells renders cells susceptible to JAK inhibition.

**Figure 6:**
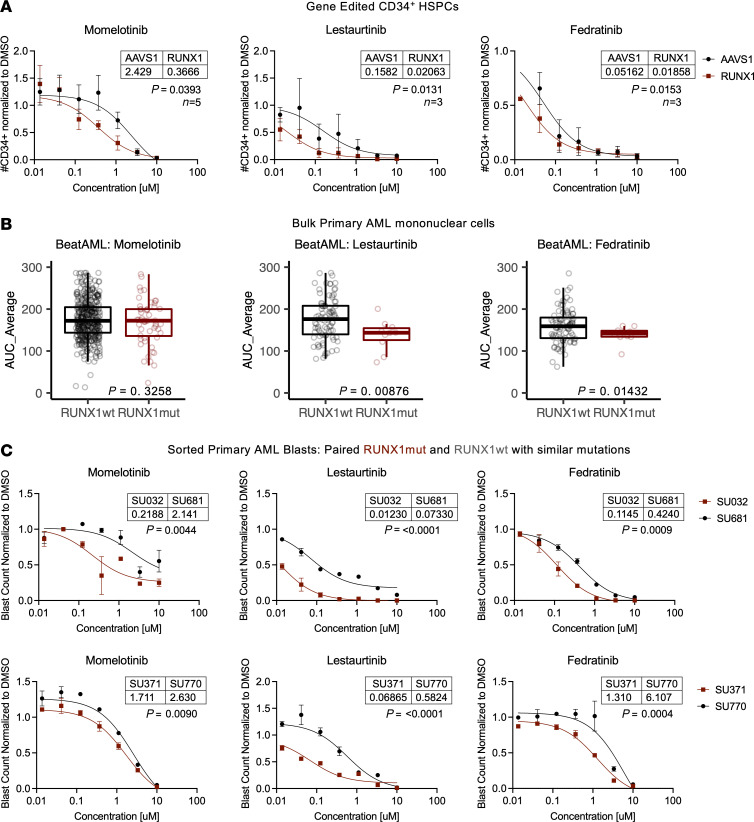
RUNX1 loss sensitizes HSPCs and primary AML blasts to JAK inhibition. (**A**) CD34^+^ HDR HSPCs were treated with JAK inhibitors or DMSO control for 72 hours, and cell numbers were determined using CountBright beads and flow cytometry. *n* = 3–5 CB. Least sum-of-squares *F* test. (**B**) Drug sensitivity to JAK inhibitors in RUNX1wt and RUNX1mut primary AML samples from BeatAML. *n* = 87–406 RUNX1wt, *n* = 11–53 RUNX1mut. Unpaired *t* test. (**C**) RUNX1mut (SU032, SU371, dark red) and RUNX1wt (SU681, SU770, black) were treated with JAK inhibitors or DMSO control for 72 hours, and cell numbers were determined using CountBright beads and flow cytometry. *n* = 2 technical replicates. Least sum-of-squares *F* test.

We reasoned that decreased JAK/STAT activity and dependence on IL-3 may sensitize RUNX1-KO cells to further JAK inhibition. Treatment with multiple JAK inhibitors for 72 hours preferentially reduced RUNX1-KO compared with AAVS1 control HSPC number (Figure 6A), indicating a potential therapeutic vulnerability.

Our previous pan-cancer analysis predicted that *RUNX1* mutations and JAK2 may have a synthetic lethal interaction (31). GSEA of published RNA-Seq data from patient cohorts indicated that *RUNX1* mutations also repressed JAK/STAT signaling in AML (Supplemental Figure 6). Thus, we evaluated whether *RUNX*1mut AML samples were more sensitive to JAK inhibition. Primary *RUNX1*mut AML bulk mononuclear cells from the BeatAML cohort showed similarly increased sensitivity to the JAK inhibitors lestaurtinib and fedratinib (Figure 6B). Finally, to validate that JAK inhibitors preferentially kill *RUNX1*mut AML blasts, we treated our previously identified matched *RUNX1*mut and *RUNX1*wt AML blasts for 72 hours with drug or DMSO control. As predicted, *RUNX1*mut status conferred preferential therapeutic vulnerability to all 3 JAK inhibitors (Figure 6C). Therefore, RUNX1 loss sensitized cells to JAK inhibition, and targeted use of JAK inhibitors may be beneficial in treating *RUNX1*mut hematopoietic disease.

## Discussion

In summary, we establish a human model of RUNX1-deficient HSPCs to investigate disease pathogenesis in patients with *RUNX1* mutations. We found that RUNX1 loss severely limited HSPC proliferation due to decreased JAK/STAT signaling, mediated in part by increased SOCS3 expression. However, increased IL-3 receptor expression enabled IL-3 to rescue RUNX1-deficient cell proliferative defects in vitro and competitive engraftment defects in vivo, augmenting phosphorylation of STAT5 to WT levels. These effects sensitized RUNX1-deficient cells to JAK/STAT inhibition, suggesting a potential therapeutic approach to RUNX1-deficient diseases.

The dysregulated JAK/STAT signaling, compensatory upregulation of IL3RA, and sensitivity to JAK inhibitors we have described was conserved across disease stages, and the findings open potential new lines of investigation for the understanding and treatment of *RUNX1*mut diseases. For example, multiple JAK inhibitors are approved for the treatment of autoimmune diseases as well as hematopoietic malignancies (26, 32), and autoimmune diseases commonly present in both RUNX1-FPD and MDS patients. In addition to investigating the use of JAK inhibitors for *RUNX1*mut AML, further studies using preclinical models or meta-analysis of electronic health records will be needed to determine whether selective or pan-JAK inhibitors can effectively treat autoimmune conditions, eradicating blasts or slowing progression without exacerbating existing cytopenias and platelet defects. Additionally, as IL-3 is non-cross-reactive in mouse and human — and mouse and human IL-3 receptor complexes are distinct in their binding affinity and cell expression (28, 33) — the consequences of IL-3 hypersensitivity and IL-3RA upregulation in *RUNX1*mut cells must be studied in the context of a human experimental system or using patient data. Of note, human plasmacytoid DCs (pDCs) are in part defined by their expression of the high-affinity IL-3RA, whereas mouse pDCs do not express the IL-3 receptor (33). Further, pDCs are associated with skin autoimmune conditions, which raises the possibility that the aberrant activation of pDCs may be contributing to the high incidence of eczema and psoriasis observed in RUNX1-FPD patients (8). Last, IL-3 is upregulated during infection, autoimmune disease, allergies, and AML (34–38), and the chronic inflammatory conditions in germline *RUNX1* disease may elevate basal IL-3 levels. Still, much remains to be determined about the regulation and expression of IL-3 in patients with *RUNX1* mutations, including whether IL-3 is elevated, which *RUNX1* mutant cells secrete IL-3, and what may trigger IL-3 expression. Characterizing the cytokine milieu and cellular players contributing to inflammation will help determine whether and when targeting IL-3/JAK/STAT is therapeutically actionable at different stages of disease associated with RUNX1 loss.

Here we focus on the potential of environmental factors, namely cytokines that signal through JAK/STAT pathways, that can overcome the RUNX1-KO proliferative defect. In the context of AML, it is likely that the acquisition of secondary mutations during disease progression can also augment growth. The importance of overcoming decreased JAK/STAT signaling is highlighted by the prevalence of mutations in the JAK/STAT pathway in RUNX1-FPD patients who progress to AML. In a survey of 172 *RUNX1*mut AML cases, the activating mutation JAK2^V617F^ occurred more frequently in germline *RUNX1* AML cases compared with sporadic *RUNX1* AML cases, and activating mutations in *MPL* and inactivating mutations in the JAK2 inhibitor *SH2B3* (encoding the LNK protein) occurred solely in germline *RUNX1*mut AML (18). In a separate report, 3 siblings independently acquired pathogenic variants in the JAK2 signaling pathway and developed AML at 5 years of age (39). Together with these studies, our findings show how both environmental and genetic factors may converge on the same pathways to promote leukemic outgrowth.

Finally, it is possible that mutations that activate STAT5 signaling affect the efficacy of treatment with JAK inhibitors. The kinase inhibitors described in our study showed activity against JAK2 as well as FLT3–internal tandem duplication (FLT3-ITD) mutations, which can aberrantly phosphorylate STAT5 (40–42). The paired patient AML samples evaluated in this study harbored neither *JAK2* nor *FLT3-ITD* mutations (Supplemental Table 1), and further investigation with additional samples or models will be needed to evaluate which JAK inhibitors are most potent in *RUNX1*mut disease and whether the presence of STAT5-activating mutations confers additional sensitivity or resistance in combination with *RUNX1* mutations.

Overall, this study identifies a targetable mechanism by which inflammation preferentially expands competitively disadvantaged HSPCs and highlights the need to characterize the interactions between genetics and inflammation in a variety of models and cell types.

## Methods

### Primary human samples

Mononuclear cells from CB and AML samples were isolated by Ficoll-Paque PLUS (GE Healthcare) density gradient centrifugation, and red blood cells were removed by ACK lysis (ACK Lysing Buffer, Thermo Fisher Scientific). CD34^+^ HSPCs were enriched from CB mononuclear cells by magnetic cell separation using MACS CD34 MicroBeads (Miltenyi Biotec). AML samples were resuspended in 90% FBS + 10% DMSO (MilliporeSigma) and cryopreserved in liquid nitrogen for future use.

CD34-enriched cells were either cryopreserved for future use or cultured in low-density conditions (200,000 cells/mL) at 37°C in 5% CO_2_ in StemSpan SFEM II (STEMCELL Technologies) base media supplemented with 20 ng/mL TPO, SCF, FLT3L, and IL-6 (Peprotech), and 35 nM small-molecule UM-171.

### rAAV6 viral vector construction and production

AAV vector plasmids were cloned in the pAAV-MCS plasmid (Agilent Technologies) containing inverted terminal repeat (ITR) sequences from AAV serotype 2 (AAV2). Construction of the *AAVS1*-targeting recombinant AAV6 (rAAV6) has been previously described (19, 43). The left and right homology arms for the *RUNX1* locus HDR donors were 379 and 400 bp, respectively. The *SOCS3*-targeting shRNA sequences (5′-CCACCTGGACTCCTATGAGAA-3′, 5′-GAAGAGCCTATTACATCTACT-3′) were synthesized by IDT and cloned into the pRSI9 DECIPHER shRNA expression lentiviral plasmid (AddGene) using restriction enzyme cloning, as previously described (44). The pRIS9 vector containing scramble control shRNA (5′-CACTACCAGAGCTAACTCAGATAGTACT-3′) was previously published (45). The U6-shRNA sequence was subsequently cloned into the *AAVS1*- and *RUNX1*-targeting vector plasmids. Knockout of RUNX1 protein by *RUNX1*-targeting rAAV6 vectors and knockdown of SOCS3 by *SOCS3*-targeting shRNA constructs were determined using Western blot analysis.

rAAV6 was either purchased from Vigene Biosciences Inc. or produced in HEK293FT cells. Briefly, HEK293FT cells were cotransfected using polyethylenimine with 6 μg ITR-containing plasmid and 22 μg pDGM6 packaging vector containing the AAV6 cap genes, AAV2 rep genes, and adenovirus 5 helper genes. rAAV6 was harvested after 3 days using the AAVpro Purification Kit (Takara Bio Inc.) according to the manufacturer’s instructions and then stored at –80°C until further use.

### CRISPR/Cas9 editing of primary HSPCs

CRISPR/Cas9 nucleofection and rAAV6-mediated HDR were performed as previously described (19, 43). Synthetic, chemically modified sgRNA targeting *RUNX1* (5′-UACCUUGAAAGCGAUGGGCA-3′) and *CCR5* (5′-GCAGCAUAGUGAGCCCCAGAA-3′) (25) and Cas9 protein (Alt-R HiFi CRISPR-Cas9) were purchased from Integrated DNA Technologies; sgRNAs targeting *SOCS3* was purchased from Synthego as part of the CRISPR gene knockout kit (5′-CAGCAGGUUCGCCUCGCCGC-3′; 5′-GCACUGCGUUCACCACCAGC-3′; 5′-CAGGGGGCGGCUCAUCCCGG-3′). sgRNA was precomplexed with Cas9 protein at a molar ratio of 1:2.5 at 25°C for 10 minutes immediately prior to electroporation into HSPCs. HSPCs were electroporated 2 days after isolation using the Lonza Nucleofector 4D (program DZ-100) at 5 × 10^6^ cells/mL and 150 μg/mL Cas9 protein in P3 Primary Cell Nucleofector Solution with 1× Supplement 1 (Lonza). For rAAV6-mediated HDR, rAAV6 donor vectors were added at a multiplicity of infection of 50,000 vector genomes/cell following electroporation, and cells were cultured for 3 days (including the 8-hour incubation with rAAV6 after electroporation) before isolation of CD34^+^ fluorescent protein double-positive populations by FACS using a FACSAria II SORP or FACSAria Fusion SORP (BD) for further experiments.

### Flow cytometry/FACS

Cells were washed with FACS buffer (PBS, 2% FBS, 2 mM EDTA) and stained with antibodies for 30 minutes at 4°C in less than 1 × 10^6^/50 μL total volume. For viability staining, cells were stained with propidium iodide (Life Technologies) at a final concentration of 1 mg/mL or DAPI (Thermo Fisher Scientific) at a final concentration of 0.1 mg/mL immediately prior to analysis or sorting; or cells were stained with Ghost Dye Red 780 (Tonbo) for 30 minutes at 4°C in PBS prior to antibody staining. Intracellular staining was performed using the Cytofix/Cytoperm Kit (BD Biosciences). All cell-sorting steps were validated using post-sort analyses to verify the purity of the sorted cell populations. All antibodies used for flow cytometry are detailed in Supplemental Table 2. Flow-cytometry analysis and FACS were performed on a CytoFLEX flow cytometer (Beckman Coulter), FACSymphony A5 (BD), FACSAria II SORP (BD), and FACSAria Fusion SORP (BD).

### Genomic PCR

Genomic DNA from 1 × 10^4^ to 1 × 10^5^ cells was extracted using QuickExtract DNA Extraction Solution (Lucigen) according to the manufacturer’s instructions and stored at –20°C until further use. In/out genomic PCR to validate HDR of rAAV6 in the *RUNX1* locus was performed using the following primer pair: 5′-AGCCCATCCTGGGTCAGAGG-3′, 5′-CGCGCTGAAGTCTCCGGCTA-3′.

### Quantitative PCR

Total RNA was extracted from 100,000–200,000 edited CB-derived HSPCs using the QIAGEN RNeasy Micro Kit following the manufacturer’s protocol and reverse transcribed using SuperScript III Reverse Transcriptase (Thermo Fisher Scientific).

### Western blot analysis

Whole-cell protein lysates were obtained from 1 × 10^6^ to 3 × 10^6^ HSPCs in RIPA buffer (150 mM NaCl, 0.1% Triton X-100, 0.5% sodium deoxycholate, 0.1% SDS, 50 mM Tris-HCl [pH 8.0]) and 1× Halt Protease Inhibitor Cocktail (Thermo Fisher Scientific) after centrifugation at 177*g* at 4°C for 30 minutes. Protein concentration was determined with a Pierce BCA Protein Assay Kit (Thermo Fisher Scientific) according to the manufacturer’s instructions. Lysates were denatured in NuPAGE LDS sample buffer (Thermo Fisher Scientific) supplemented with 5% β-mercaptoethanol by incubation at 95°C for 3 minutes. Denatured samples were loaded onto a NuPAGE 4-12% Bis-Tris polyacrylamide gel (Thermo Fisher Scientific), electrophoresed in MOPS running buffer (Invitrogen), and transferred onto 0.45 mm nitrocellulose membranes (Amersham Protran, GE Healthcare Life Sciences) with the Bio-Rad Mini-PROTEAN Tetra wet tank transfer system. Membranes were blocked with 5% wt/vol milk for either 2 hours at room temperature or overnight before staining with primary antibodies. Primary antibodies utilized were anti-RUNX1 rabbit monoclonal antibody (D33G6, Cell Signaling Technology, 433G), anti-Vinculin mouse monoclonal antibody (V284, Bio-Rad, MCA465GA), and anti-SOCS3 mouse monoclonal antibody (1B2, Thermo Fisher Scientific, 37-7200). Clarity Western ECL Substrate (Bio-Rad) was used to detect antibodies after staining with HRP-conjugated secondary antibodies (anti-rabbit, Cell Signaling Technology, 7074; or anti-mouse, Cell Signaling Technology, 7076) or both biotin-conjugated secondary antibodies (anti-rabbit, Invitrogen, 31820; or anti-mouse, Invitrogen, 31800) and HRP-conjugated streptavidin (Thermo Fisher Scientific). Membranes were imaged either using Amersham Hyperfilm ECL high-performance chemiluminescence film (GE Healthcare) or with the Bio-Rad GelDoc system.

### In vitro liquid differentiation and colony assays

For liquid differentiation assays, CD34^+^ HDR HSPCs were plated in megakaryocyte differentiation media (StemSpan SFEM II supplemented with 40 mg/mL human LDLs and 1× StemSpan Megakaryocyte Expansion Supplement [STEMCELL Technologies]), erythroid differentiation media (StemSpan SFEM II supplemented with 1× StemSpan Erythroid Expansion Supplement [STEMCELL Technologies]), or myeloid differentiation media (MyeloCult H5100 [STEMCELL Technologies]) supplemented with 20 ng/mL SCF, FLT3L, IL-3, IL-6, GM-CSF, and G-CSF [Peprotech] and 0.5 μg/mL hydrocortisone). Cells were cultured for 7 days, with media changes every 2 or 3 days, and liquid differentiation assays were read out by flow cytometry using either a FACSAria II SORP or CytoFLEX flow cytometer system, with appropriate antibody staining as indicated.

For colony formation assays, 2,000–4,000 CD34^+^ HSPCs in 400 μL IMDM were added to 4 mL MethoCult H4435 (STEMCELL Technologies), and 1.1 mL was plated in triplicate (500–1,000 cells/plate). The number and type of colonies in each sample were determined at 14 days by morphology. For collagen-based megakaryocyte-colony forming assays, 10,000 CD34^+^ HSPCs in 400 μL IMDM were added to MegaCult (STEMCELL Technologies) supplemented with 600 μL collagen and 50 ng/mL TPO, 10 ng/mL IL-6, and 10 ng/mL IL-3 (Peprotech). 750 μL was plated in duplicate (5,000 cells/well) on double-chamber slides, and the number of colonies in each sample was scored at 10 days.

### In vitro proliferation assays and cytokine treatment

Cells were cultured in stem retention media, SFEM II, supplemented with 20 ng/mL TPO, SCF, FLT3L (Peprotech), with or without additional cytokines (IL-1A, IL-1B, IL-3, IFN-β, IFN-γ, GM-CSF, G-CSF, TNF-α [Peprotech]) or LPS (Invitrogen). After 6 days of culture, cell count was determined by flow cytometry using CountBright absolute counting beads (Invitrogen).

### In vitro cell-cycle, apoptosis, and CellTrace assays

Cell-cycle analysis was performed using the Click-iT EdU Alexa Fluor 647 Flow Cytometry Assay Kit (Invitrogen) following the manufacturer’s protocol. Briefly, cells cultured in stem retention media were incubated with 1 μM EdU at 37°C for 2 hours, fixed at room temperature for 15 minutes, permeabilized at room temperature for 15 minutes, incubated with the Click-iT reaction cocktail at room temperature for 30 minutes, and stained with DAPI at a final concentration of 0.2 mg/mL.

For apoptosis, cells were washed twice with annexin binding buffer (10 nM HEPES, 140 nM NaCl, 2.5 mM CaCl_2_, pH 7.4) and stained with Annexin V Alexa Fluor 647 (1:50; A23204, Thermo Fisher Scientific) and DAPI at room temperature for 15 minutes.

For CellTrace Assays, cells were labeled with 5 μM CellTrace Violet (CellTrace Violet Proliferation Kit, Invitrogen) in PBS at 37°C for 20 minutes, incubated in 5× volume stem retention media for at 37°C for 5 minutes, washed, and cultured for 4 days before analysis by flow cytometry.

### Animal studies

Animal experiments were performed with NOD.Cg-Prkdc^scid^ Il2rg^tm1Wjl^/SzJ (NSG) or NOD.Cg-Prkdc^scid^ Il2rg^tm1Wjl^Tg(CMV-IL3,CSF2,KITLG)1Eav/MloySzJ (NSGS) mice purchased from The Jackson Laboratory and bred in-house. Mice were housed in specific pathogen–free animal facilities in microisolator cages.

Human cells were engrafted in 4- to 12-week-old male or female mice 2–24 hours after sublethal irradiation (100–200 rad) by intrafemoral injection of 50,000–300,000 cells. Every 8–10 weeks after transplantation, bone marrow aspirates were obtained from the transplanted femur. For terminal engraftment analysis, mice were humanely euthanized, and femurs, tibias, hip bones, sternum, and spine were harvested and crushed. Mononuclear cells were isolated by Ficoll-Paque PLUS density gradient centrifugation. For both bone marrow aspirate and terminal engraftment analysis, red blood cells were removed with ACK lysis (ACK Lysing Buffer), and remaining cells were stained for flow cytometry.

### RNA-Seq

#### Library preparation and sequencing.

CD34^+^ HDR HSPCs were sorted 3 days after editing, expanded for 7 days, and re-sorted on CD34 and HDR positivity for RNA-Seq. Total RNA was extracted from 100,000–200,000 edited CD34^+^ HDR HSPCs using the QIAGEN RNeasy Micro Kit following the manufacturer’s protocol. RNA quality control, library preparation, Illumina Next Generation Sequencing, and data quality control were performed at the Novogene commercial sequencing facility. All sequenced samples had an RNA integrity number (RIN) greater than 7.7. Briefly, for RNA-Seq library preparation, mRNA was enriched using oligo-dT–coupled magnetic beads. First-strand cDNA synthesis was performed using random hexamers; this was followed by second-strand cDNA synthesis, terminal repair, 3′A-tailing, adapter ligation, size selection, and PCR amplification. Between 20 and 28 million paired-end sequencing reads per sample were delivered by Novogene.

#### Analysis.

FASTQ sequencing reads were trimmed and quality checked using fastp (46) and quantified using kallisto (47) pseudoalignment to a cDNA index built using gene annotations downloaded from Ensembl and the reference human genome GRCh38. The kallisto output was imported into R using tximport (48) using gene mode. Differential testing was performed using DESeq2 (49) with CB donor as cofactor. GSEA was performed using the GSEA Mac App v4.2.3 (Broad Institute Inc.). Volcano plots and dot plots were generated using ggplot2 (50); heatmaps were generated using pheatmap (51).

### ATAC-Seq

#### Library preparation and sequencing.

CD34^+^ HDR HSPCs were sorted 3 days after editing for ATAC-Seq. Two technical ATAC-Seq library replicates from each CD34^+^ HDR HSPC sample were prepared as previously described (52). Briefly, 10,000 cells were washed in FACS buffer at 4°C and spun down. Cell pellets were then resuspended in 50 μL ATAC-Seq resuspension buffer (RSB: 10 mM Tris-HCl [pH 7.4], 10 mM NaCl, and 3 mM MgCl_2_ in water) with 0.1% NP40, 0.01% digitonin, and 0.1% Tween-20, and incubated on ice for 3 minutes. After lysis, 1 mL ATAC-Seq RSB with 0.1% Tween-20 was added, and tubes were inverted 6 times to mix. Isolated nuclei were then spun down. Supernatant was removed, and nuclei were resuspended in 50 μL transposition mix (25 μL 2×TD buffer, 2.5 μL Tn5 transposase [100 nM final], 16.5 μL PBS, 0.5 μL 1% digitonin, 0.5 μL 10% Tween-20, and 5 μL nuclease-free water). Transposition reactions were incubated at 37°C for 30 minutes in a thermomixer with shaking at 1,000 rpm. Reactions were cleaned up using QIAGEN MinElute Reaction Cleanup Kits and processed as previously described (52). All libraries were amplified with a target concentration of 20 μL at 4 nM, which is equivalent to 80 femtomoles of product, and submitted to Novogene for sequencing on an Illumina NovaSeq platform with 2 × 150 read configuration.

#### Preprocessing, peak calling and merging, and count matrix generation.

ATAC-Seq data were processed based on workflows derived from Corces, Granja, et al. (53). First, read trimming and quality control were performed using trim_galore (54) using default parameters. Reads were aligned to the GRCh38 reference genome using bowtie2 (55) and the “—very-sensitive” option. Aligned reads were converted to BAM format and sorted using SAMtools (56), and deduplicated using Picard MarkDuplicates (57). Reads were subsequently indexed, and mitochondrial reads were removed using SAMTools (56). TagAlign bed files from technical replicate libraries were generated using bedtools (58), shifted to find ATAC-Seq cut sites, and pooled. Peaks were called using MACS2 (59) using a *P* value cutoff of 0.01, and peaks falling within black list regions (https://www.encodeproject.org/annotations/ENCSR636HFF/) were removed. A consensus peak set was generated by concatenating peaks of all samples into a single bed file and merged using the ArchR Create Extended PeakSet script. Peaks extending past chromosome ends and peaks that mapped to chrY were excluded. Count matrices were generated using the consensus peak set, and the number of reads in each peak were calculated using the CountOverlaps function from the Iranges (60) package in R software.

#### Chromatin accessibility analysis.

Differential testing was performed using DESeq2 (49) with CB donor included in the differential testing model as a covariate to control for between-cord effects. Peaks were annotated using the annotatePeak function from the ChIPseeker (61) package and the UCSC hg38 reference. Distal enhancer regions were further defined as previously described (62). In brief, we merged overlapping and close (<5 kb) distal regions from our consensus peak set with H3K27ac ChIP-Seq data from CD34^+^ common myeloid progenitors (ENCODE experiment ENCSR891KSP) and extended these regions by 1 kb. GSEA was performed using the GSEA Mac App v4.2.3 (Broad Institute Inc.). Transcription factor motif enrichment was performed using ChromVAR (63) as previously described using JASPAR 2018 motif clusters (64) to reduce redundancy. Volcano plots and dot plots were generated using ggplot2 (50); heatmaps were generated using pheatmap (51).

### ChIP-Seq processing and analysis

Bed files from the National Center for Biotechnology Information Gene Expression Omnibus database (GEO GSE45144) were downloaded, and peaks were called using MACS2 (59) using the *q* value cutoff of 0.05. Peaks were annotated using the annotatePeak function from the ChIPseeker (61) package and the UCSC hg19 reference genome. To increase stringency, peaks were filtered to retain peaks <1 kb distance from transcriptome start sites for analysis. Genome tracks were visualized with Integrative Genomics Viewer v2.8.9 (65).

### In vitro drug experiments

NF-κB pathway and JAK inhibitors were dissolved in DMSO and serially diluted in DMSO. 1,000–5,000 sorted CD34^+^ HDR HSPCs were seeded in 96-well culture plates with a final volume of 200 μL myeloid differentiation media for NF-κB inhibitors, or stem retention culture media for JAK inhibitors, 24 hours prior to the addition of inhibitor. For primary AML blast drug assays, 50,000 cells were seeded in 96-well culture plates in a final volume of 100 μL stem retention culture media 6–8 hours prior to the addition of JAK inhibitor. Inhibitors or DMSO control were diluted in culture media and were added to on day 1 for a final concentration of 0.1% DMSO and inhibitor concentrations as indicated. For NF-κB pathway inhibitor experiments, media was changed after 3 days, and myeloid differentiation was read out by flow cytometry 6 days after addition of drug. For JAK inhibitor experiments, cell viability and cell count were determined 72 hours after addition of drug by flow cytometry using CountBright absolute counting beads.

### Statistics

Statistical analyses were performed in R version 4.0.1 or Prism9 (GraphPad Software). Paired and unpaired 2-tailed *t* test was used to define statistical significance. A *P* value of less than 0.05 was considered significant. One-way or 2-way ANOVA tests and the appropriate multiple-comparison test were performed for experiments with more than 2 conditions. Experiments were performed with at least 3 biological replicates, with technical duplicates or triplicates per biological sample unless otherwise noted. Data represent mean ± SEM unless otherwise noted.

### Study approval

#### Human samples.

Umbilical CB was collected with written informed consent from the mother before delivery at the Lucile Packard Children’s Hospital through the Stanford Binns Program for Cord Blood Research (Stanford University IRB 33818), purchased from the New York Blood Center, or purchased from the Carolinas Cord Blood Bank. Adult mobilized peripheral blood was purchased from the Fred Hutchinson Cancer Center. AML patient samples were obtained from AML patients with written informed consent according to Administrative Panel on Human Subjects Research IRB–approved protocols (Stanford University IRB 6453).

#### Animal studies.

All mouse experiments were conducted in accordance with a protocol approved by the Institutional Animal Care and Use Committee (Stanford Administrative Panel on Laboratory Animal Care 22264) and in adherence with the NIH *Guide for the Care and Use of Laboratory Animals* (National Academies Press, 2011).

### Data availability

RNA-Seq and ATAC-Seq data are available in the NCBI GEO database (GSE231951). RUNX1 ChIP-Seq data were downloaded from the NCBI GEO database (GSE45144). H3K37ac ChIP-seq data were downloaded from the ENCODE database (experiment ENCSR891KSP). BeatAML RNA-Seq and drug response data were downloaded from the BeatAML vizome interface (http://vizome.org/aml), and TCGA RNA-Seq data were downloaded from cBioPortal for Cancer Genomics (http://www.cbioportal.org). Values for all data points in graphs are reported in the Supporting Data Values file.

## Author contributions

ACF and RM conceived of the work. ACF, YN, KAN, FZ, DK, DCH, and AR performed the experimental work. ACF, LB, AA, and TK performed computational analysis. ACF and RM wrote the manuscript with input from all authors. PK and RM supervised the research.

## Supplementary Material

Supplemental data

Supporting data values

## Version 1 08/15/2023

In-Press Preview

## Version 2 10/02/2023

Electronic publication
