# Supplementary material for: RUNX1 loss renders hematopoietic and leukemic cells dependent on IL-3 and sensitive to JAK inhibition
Source: J Clin Invest. 2023 Oct 2;133(19):e167053. doi: 10.1172/JCI167053 (PMC10541186; doi:10.1172/JCI167053)
Supplement: Supplemental data [file jci-133-167053-s030.pdf]

Supplemental material

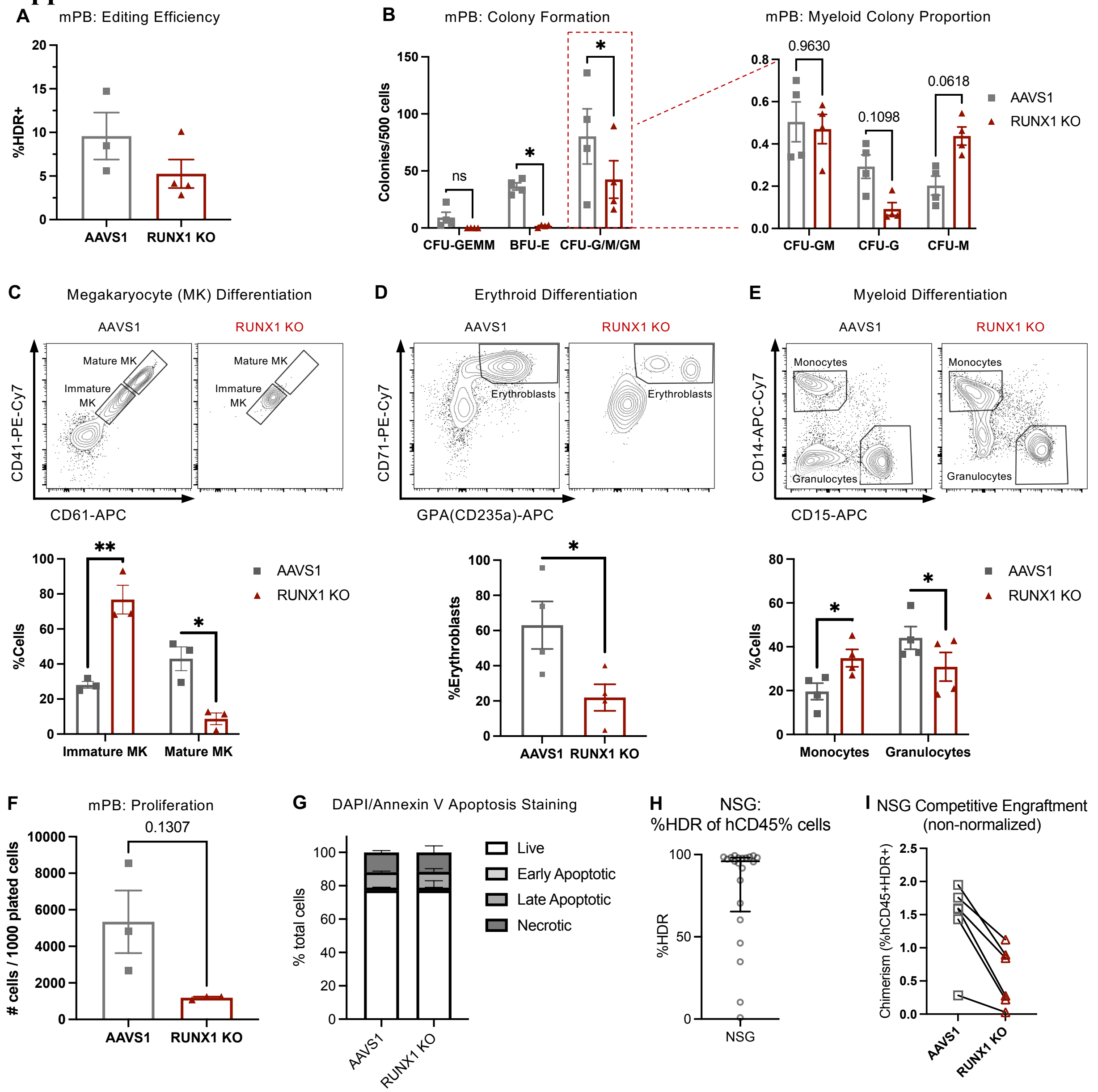

Supplemental Figure 1: RUNX1 loss in human HSPCs expands monocytic cells at the expense of erythro-megakaryocytic differentiation.
(A) Quantification of double positive HDR editing efficiency at *AAVS1* safe harbor locus and *RUNX1* locus in CD34$^+$ HSPCs. n = 3-4 adult mobilized peripheral blood (mPB).
(B) CD34$^+$ HDR HSPCs from mPB were plated in methocellulose-based colony forming assays and assessed for colony formation at 14 days. n = 4 mPB. Two-Way ANOVA, Sidak's multiple comparison's test: n.s. not significant, * p<0.05.
(C) CD34$^+$ HDR HSPCs were plated in megakaryocyte (MK) differentiation media and evaluated for CD41$^+$CD61$^+$ immature MK and CD41^{++}CD61^{++} mature MK by flow cytometry after 7 days. n = 3 CB. Two-way ANOVA, Sidak's multiple comparisons test: * p < 0.05, ** p < 0.01.

(D) $CD34^+$ HDR HSPCs were plated in erythroid differentiation media and evaluated for $CD71^+GPA^+$ erythroblasts by flow cytometry after 7 days. n = 4 CB. Paired t-test: * $p < 0.05$.
(E) $CD34^+$ HDR HSPCs were plated in myeloid differentiation media and evaluated for $CD14^+$ monocytes and $CD15^+$ granulocytes by flow cytometry after 7 days. n = 4 CB. Two-way ANOVA, Sidak's multiple comparisons test: * $p < 0.05$.
(F) $CD34^+$ HDR HSPCs were plated in stem retention media and analyzed by flow cytometry for cell count at days 6. n = 3 CB. Paired t-test.
(G) $CD34^+$ HDR HSPCs were stained for Annexin V and DAPI. Live (Annexin V- DAPI-), Early Apoptotic (Annexin V+ DAPI-), Late Apoptotic (Annexin V+ DAPI+), and Necrotic (Annexin V-, DAPI+) cells were quantified with flow cytometry. Paired t-test: n.s. not significant. n = 2 CB.
(H) %HDR positivity in $hCD45^+$ cells upon sacrifice in NSG mice. n = 21 mice. Shown are median +/- interquartile range. Median = 95.9%.
(I) AAVS1 and RUNX1 KO cells were injected in a 1:1 ratio intrafemorally into sublethally irradiated NSGS mice and chimerism (%$hCD45^+HDR^+$) at 18 weeks was ascertained using bone marrow aspirates. n = 3 CB, 6 mice.

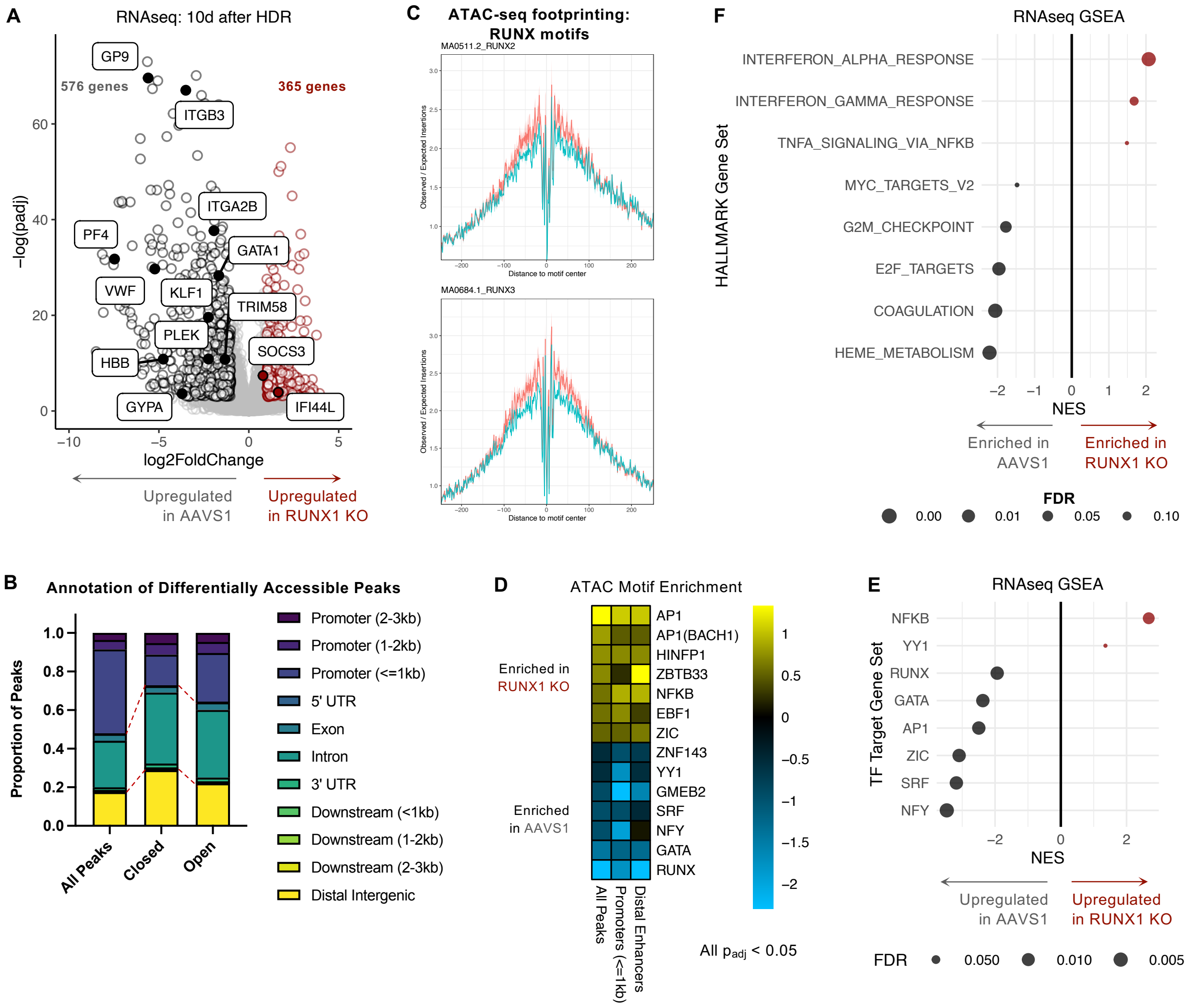

Supplemental Figure 2: RUNX1 KO causes upregulation of myeloid and inflammatory programs and downregulation of erythro-megakaryocytic programs.
(A) Volcano plot of adjusted p-valued (padj) and log2(Fold Change) of RNA-seq of $CD34^+$ HDR HSPCs 10 days after editing. Black circles, genes upregulated in AAVS1 control relative to RUNX1 KO, padj < 0.05, Log2(FC) < -1. Dark red circles, genes upregulated in RUNX1 KO relative to AAVS1, padj < 0.05, Log2(FC) > 1. Filled circles indicate highlighted genes.
(B) Annotation of all peaks and differentially open and closed peaks in ATAC-seq of $CD34^+$ HDR HSPCs 3 days after editing.
(C) Representative footprinting analysis of RUNX motifs in ATAC-seq data.
(D) Change in ATAC-seq peak transcription factor motif enrichment in RUNX1 KO cells relative to AAVS1 controls in all differentially accessible ATAC-seq peaks, promoters, and distal enhancers (based on H3K27ac loci identified in $CD34^+$ CMPs).
(E) RNA-seq GSEA of transcription factor target gene sets.
(F) RNA-seq HALLMARK gene set enrichment analysis (GSEA).

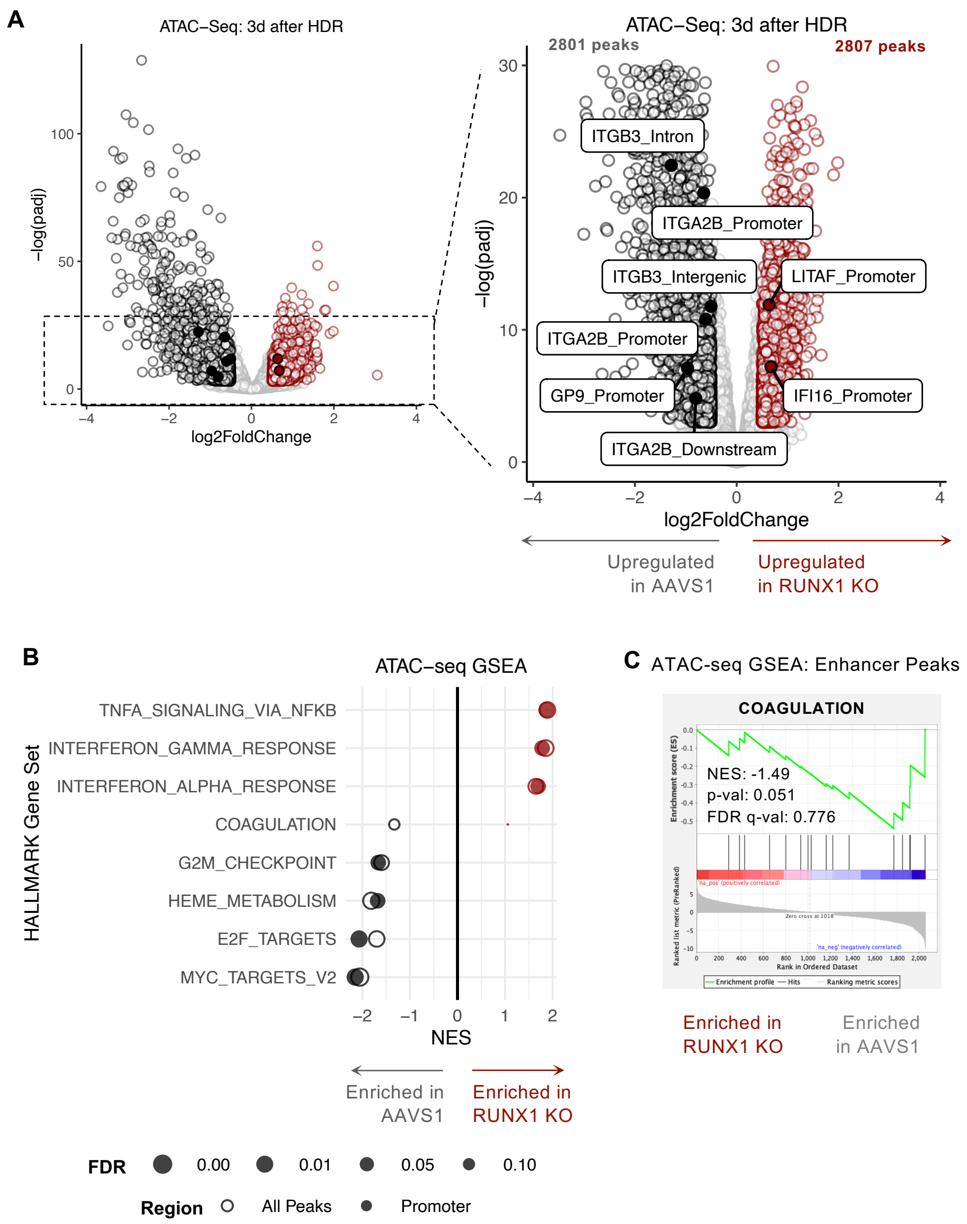

Supplemental Figure 3: Differential peak and motif accessibility in RUNX1 KO HSPCs

(A) Volcano plot of adjusted p-valued (padj) and log2(Fold Change) of ATAC-seq of $CD34^{+}$ HDR HSPCs. Black circles, peaks open in AAVS1 control relative to RUNX1 KO, padj < 0.05. Dark red circles, peaks open in RUNX1 KO relative to AAVS1, padj < 0.05. Filled circles indicate highlighted peaks.

(B) ATAC-seq HALLMARK GSEA of all differentially accessible peaks and promoter (<=1kb) peaks.

(C) ATAC-seq GSEA of HALLMARK Coagulation gene set in enhancer peaks.

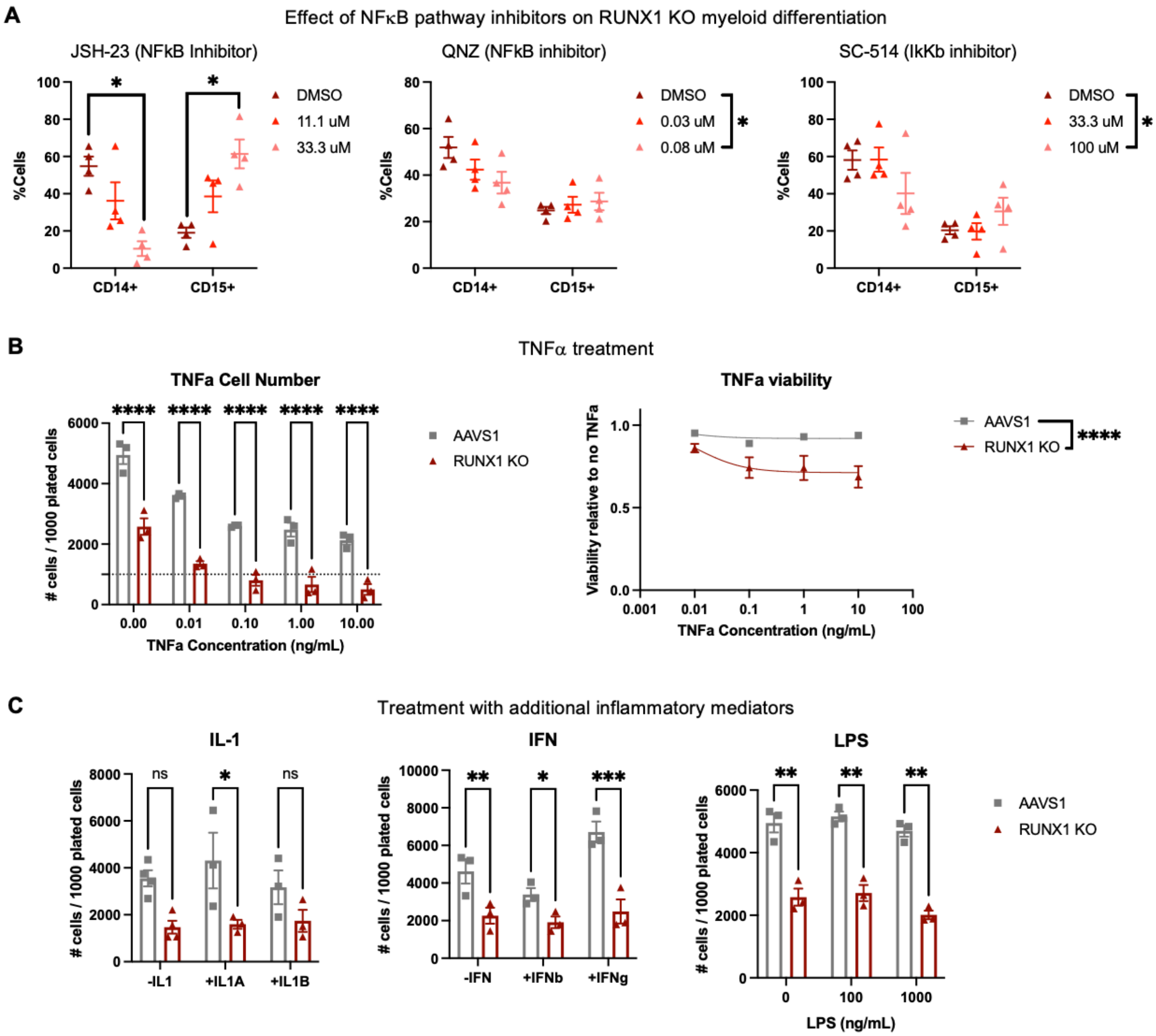

Supplemental Figure 4: Increased NFκB activity does not protect provide survival or growth advantage in inflammatory conditions

(A) RUNX1 KO cells were plated in liquid myeloid differentiation media and treated with inhibitor or DMSO control. Proportion of $CD14^+$ monocytes and $CD15^+$ granulocytes was assessed by flow cytometry at 7 days. n = 4 CB. Two-way ANOVA, Sidak's multiple comparisons test: * $p < 0.05$.

(B) $CD34^+$ HDR HSPCs were plated in stem retention media and supplemented with indicated doses of TNFα. Cell count and viability was determined at 6 days by flow cytometry. n = 3 CB. Two-way ANOVA, Sidak's multiple comparisons test or non-linear regression comparison of fits: **** $p < 0.0001$.

(C) $CD34^+$ HDR HSPCs were plated in stem retention media and supplemented with 10ng/mL IL-1A, 10 ng/mL, 1U/µL IFNβ, 1U/µL IFNγ, 100 ng/mL LPS, or 1000 ng/mL LPS. n = 3 CB. Two-way ANOVA, Sidak's multiple comparisons test: n.s. not significant, * $p < 0.05$, ** $p < 0.01$, *** $p < 0.001$.

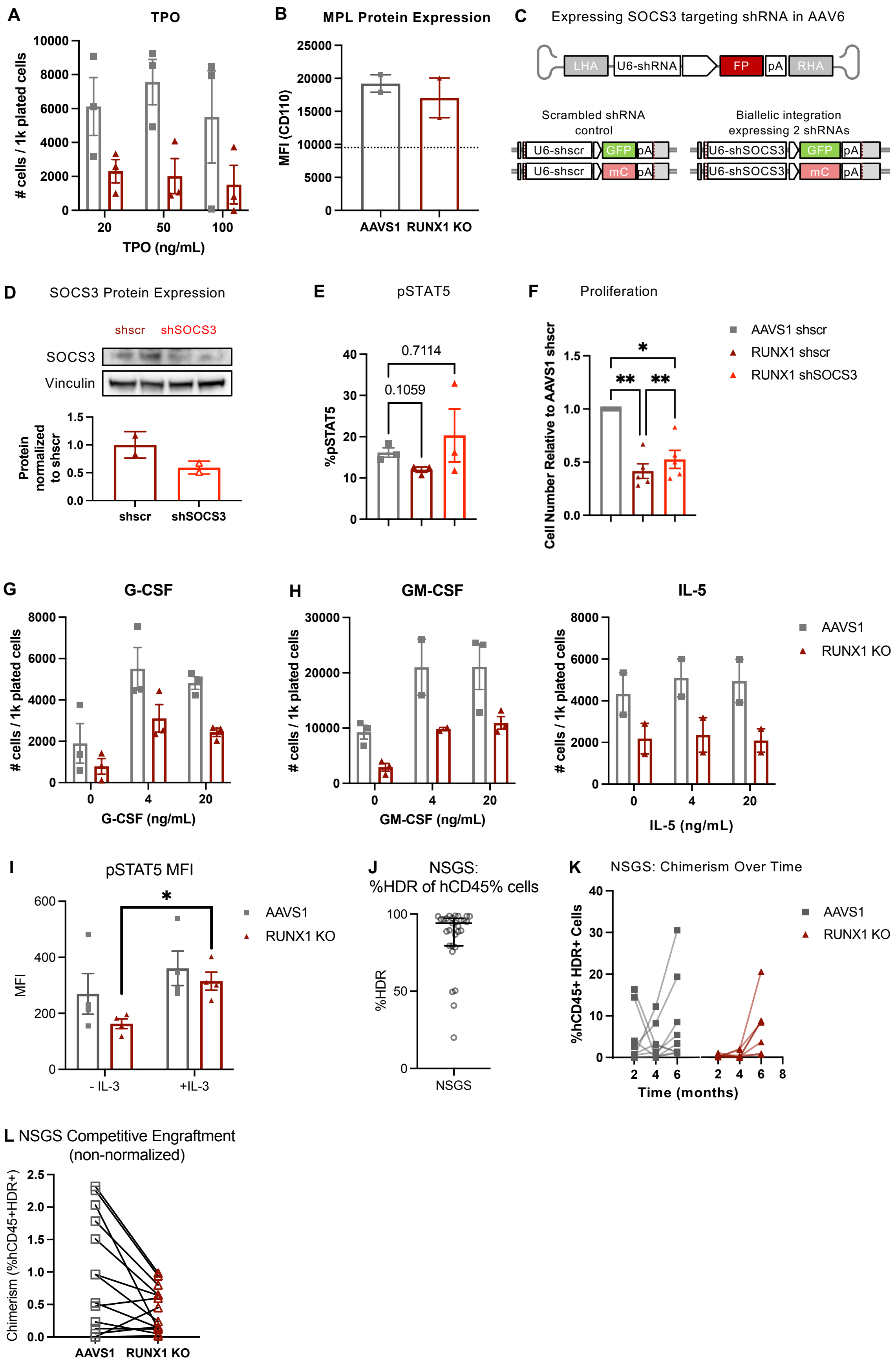

A
TPO
cells / 1k plated cells
TPO (ng/mL)
B
MPL Protein Expression
MFI (CD110)
AAVS1
RUNX1 KO
C
Expressing SOCS3 targeting shRNA in AAV6
LHA
U6-shRNA
FP
pA
RHA
Scrambled shRNA control
Biallelic integration expressing 2 shRNAs
U6-shscr
U6-shSOCS3
GFP
mC
D
SOCS3 Protein Expression
shscr
shSOCS3
SOCS3
Vinculin
Protein normalized to shscr
E
pSTAT5
%pSTAT5
0.7114
0.1059
F
Proliferation
Cell Number Relative to AAVS1 shscr
AAVS1 shscr
RUNX1 shscr
RUNX1 shSOCS3
G
G-CSF
G-CSF (ng/mL)
H
GM-CSF
GM-CSF (ng/mL)
IL-5
IL-5 (ng/mL)
AAVS1
RUNX1 KO
I
pSTAT5 MFI
MFI
- IL-3
+IL-3
J
NSGS: %HDR of hCD45% cells
%HDR
NSGS
K
NSGS: Chimerism Over Time
%hCD45+ HDR+ Cells
Time (months)
L NSGS Competitive Engraftment (non-normalized)
Chimerism (%hCD45+HDR+)

Supplemental Figure 5: Only IL-3 rescues RUNX1 KO cell proliferative defect

(A) $CD34^+$ HDR HSPCs were plated in stem retention media (20 ng/mL TPO) and supplemented with additional TPO to 50 ng/mL or 100 ng/mL. Cell count was determined at 6 days by flow cytometry. n = 3 CB.

(B) Protein expression of MPL measured by flow cytometry. n = 2 CB.

(C) Recombinant AAV6 vector carry arms of homology flanking U6-driven shRNA and fluorescent protein (FP) reporter transgenes as donor DNA for HDR. Integration of both GFP and mCherry donors will result in either expression of 2 copies of scrambled shRNA control (shscr) or 2 shRNAs targeting *SOCS3* (shSOCS3).

(D) Western blot for SOCS3 in cells with scrambled shRNA control ("shscr") or SOCS3 targeting shRNAs ("shSOCS3") integrated into the *RUNX1* locus and quantification of SOCS3 protein knockdown in shSOCS3 cells relative to shscr cells.

(E) $CD34^+$ HDR HSPCs were plated in stem retention media for 6 days and analyzed by flow cytometry for pSTAT5. pSTAT5 positivity was gated based on isotype controls. n = 3 CB. One-way ANOVA, Dunnett's multiple comparisons test.

(F) $CD34^+$ HDR HSPCs were plated in stem retention media for 6 days and analyzed by flow cytometry for cell count using CountBright beads. Cell counts normalized to AAVS1 + shscramble controls are shown. n = 6 CB. One-way ANOVA: n.s. not significant, * $p < 0.05$, ** $p < 0.01$.

(G, H) $CD34^+$ HDR HSPCs were plated in stem retention media and supplemented with G-CSF, GM-CSF, or IL-5. Cell count was determined at 6 days by flow cytometry. n = 2-3 CB.

(I) pSTAT5 MFI was quantified in $CD34^+$ HDR HSPCs plated in serum-free media with SCF, TPO, and FLT3L with or without 10 ng/mL IL-3 after 7 days. Two-way ANOVA, Sidak's multiple comparison's test: * $p < 0.05$.

(J) %HDR positivity in $hCD45^+$ cells upon sacrifice in NSGS mice. n = 29 mice. Shown are median +/- interquartile range. Median = 94.1%.

(K) $CD34^+$ HDR HSPCs were injected intrafemorally into sublethally irradiated NSGS mice and $hCD45^+HDR^+$ engraftment monitored over time using bone marrow aspirates (at 8-10 weeks or 16-18 weeks after transplantation) and upon sacrifice (at 24-26 weeks after transplantation). n = 3 CB, 16 mice.

(L) AAVS1 and RUNX1 KO cells were injected in a 1:1 ratio intrafemorally into sublethally irradiated NSGS mice and chimerism (%$hCD45^+HDR^+$) at 18 weeks was ascertained using bone marrow aspirates. n = 3 CB, 13 mice.

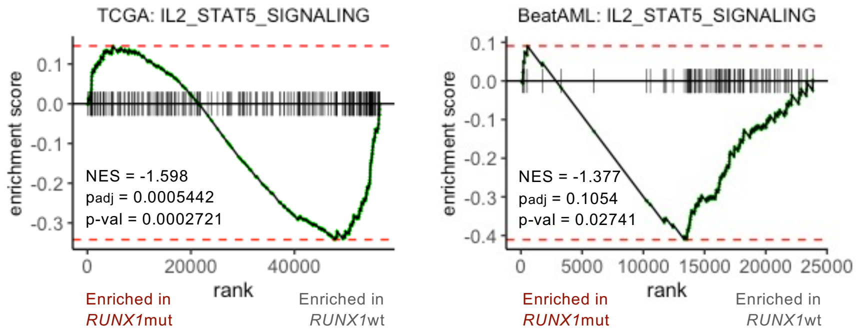

Supplemental Figure 6: STAT5 signaling is downregulated in *RUNX1*mut AMLs
IL2 STAT5 Signaling GSEA comparing *RUNX1*mut to *RUNX1*wt AMLs in TCGA AML and BeatAML datasets.

Supplementary Table 1: Patient Samples

Sample	RUNX1 Mutations (VAF)	Non-RUNX1 mutations
SU032	RUNX1^{S381A} (48.62%), RUNX1^{P103T} (5.19%)	CEBPA
SU371	RUNX1^{F353} (46.97%)	TET2, ASXL1
SU524		TET2, ASXL1
SU681		CEBPA, CSF3R
SU770		TET2, ASXL1

Supplementary Table 2: Flow Cytometry Antibodies

Antigen	Fluorophore	Clone	Supplier
CD110 (MPL)	BV421	1.6.1	BD
CD114 (CSF3R, G-CSFR)	APC	LMM741	Biolegend
CD116 (CSF2RA, GM-CSFR)	BV421	hGMCSFR-M1	BD
CD123 (IL3RA)	APC	7G3	BD
CD123 (IL3RA)	BUV395	7G3	BD
CD14	APC-Cy7	MφP9	BD
CD15	APC	MMA	eBioscience
CD19	PE-Cy5	HIB19	BD
CD20	PE-Cy5	2H7	BD
CD235a (GPA)	APC	HIR2	Biolegend
CD3	APC-Cy7	SK7	BD
CD34	APC	581	Biolegend
CD34	APC	8G12	BD
CD38	PE-Cy7	HB7	BD
CD41	PE-Cy7	HIP8	BD
CD61	APC	VIPL2	BD
CD71	PE-Cy7	OKT9	eBioscience
CD99	FITC	TÜ12	BD
human CD45	V450	HI30	BD
human CD45	APC	2D1	eBioscience
mouse CD45.1	PE-Cy7	A20	Invitrogen
Ter119	PE-Cy5	TER-119	Invitrogen
TIM-3	PE	344823	R&D Systems
pSTAT5 (pY694)	AF647	47/Stat5	BD

(e.g. 20221230)

Search results

Results for query **"(majeti).in. AND (stanford).as."** Showing 1 to 38 of 38 records

Result #	Document/Patent number		Title	Inventor name	Publication date	Pages
1	US-11718670-B2	Preview PDF	Methods for determining and achieving therapeutically effective doses of anti-CD47 agents in treatment of cancer	Weissman; Irving L. et al.	2023-08-08	29
2	US-11603404-B2	Preview PDF	Methods for treating cancer by achieving therapeutically effective doses of anti-CD47 antibody	Willingham; Stephen et al.	2023-03-14	52
3	US-11518806-B2	Preview PDF	Methods for treating cancer by achieving therapeutically effective doses of anti-CD47 antibody	Willingham; Stephen et al.	2022-12-06	51
4	US-11472878-B2	Preview PDF	Methods for determining and achieving therapeutically effective doses of anti-CD47 agents in treatment of cancer	Weissman; Irving L. et al.	2022-10-18	29
5	US-11377495-B2	Preview PDF	Markers of acute myeloid leukemia stem cells	Majeti; Ravindra et al.	2022-07-05	89
6	US-11141480-B2	Preview PDF	Dosing parameters for CD47 targeting therapies to hematologic malignancies	Majeti; Ravindra et al.	2021-10-12	26
7	US-11136391-B2	Preview PDF	Methods for treating cancer by achieving therapeutically effective doses of anti-CD47 antibody	Willingham; Stephen et al.	2021-10-05	52
8	US-11104731-B2	Preview PDF	Compositions for achieving therapeutically effective doses of anti-CD47 agents	Willingham; Stephen et al.	2021-08-31	46
9	US-11072655-B2	Preview PDF	Markers of acute myeloid leukemia stem cells	Majeti; Ravindra et al.	2021-07-27	63
10	US-11014985-B2	Preview PDF	Humanized and chimeric monoclonal antibodies to CD47	Liu; Jie et al.	2021-05-25	45
11	US-10995152-B2	Preview PDF	Modified immunoglobulin hinge regions to reduce hemagglutination	Liu; Jie et al.	2021-05-04	20

12	US-10942185-B2	Preview PDF	Therapeutic and diagnostic methods for manipulating phagocytosis through calreticulin and low density lipoprotein-related receptor	Chao; Mark P. et al.	2021-03-09	34
13	US-10662242-B2	Preview PDF	Markers of acute myeloid leukemia stem cells	Majeti; Ravindra et al.	2020-05-26	58
14	US-10640561-B2	Preview PDF	Methods for manipulating phagocytosis mediated by CD47	Jaiswal; Siddhartha et al.	2020-05-05	96
15	US-10487150-B2	Preview PDF	SIRP alpha-antibody fusion proteins	Majeti; Ravindra et al.	2019-11-26	46
16	US-10301387-B2	Preview PDF	Methods for achieving therapeutically effective doses of anti-CD47 agents	Willingham; Stephen et al.	2019-05-28	46
17	US-10087257-B2	Preview PDF	SIRP alpha-antibody fusion proteins	Majeti; Ravindra et al.	2018-10-02	46
18	US-10040862-B2	Preview PDF	Humanized and chimeric monoclonal antibodies to CD99	Liu; Jie et al.	2018-08-07	26
19	US-9796781-B2	Preview PDF	Markers of acute myeloid leukemia stem cells	Majeti; Ravindra et al.	2017-10-24	56
20	US-9765143-B2	Preview PDF	Methods for manipulating phagocytosis mediated by CD47	Jaiswal; Siddhartha et al.	2017-09-19	96
21	US-9624305-B2	Preview PDF	Methods of manipulating phagocytosis mediated by CD47	Jaiswal; Siddhartha et al.	2017-04-18	96
22	US-9623079-B2	Preview PDF	Methods for achieving therapeutically effective doses of anti-CD47 agents for treating cancer	Willingham; Stephen et al.	2017-04-18	46
23	US-9611329-B2	Preview PDF	Methods of manipulating phagocytosis mediated by CD47	Jaiswal; Siddhartha et al.	2017-04-04	95
24	US-9605076-B2	Preview PDF	Methods of manipulating phagocytosis mediated by CD47	Jaiswal; Siddhartha et al.	2017-03-28	96
25	US-	Preview	HUMANIZED AND CHIMERIC	Liu; Jie et al.	2017-02-02	26

	20170029524-A1	PDF	MONOCLONAL ANTIBODIES TO CD99			
26	US-9493575-B2	Preview PDF	Methods for manipulating phagocytosis mediated by CD47	Jaiswal; Siddhartha et al.	2016-11-15	93
27	US-9399682-B2	Preview PDF	Methods for manipulating phagocytosis mediated by CD47	Jaiswal; Siddhartha et al.	2016-07-26	95
28	US-9382320-B2	Preview PDF	Humanized and chimeric monoclonal antibodies to CD47	Liu; Jie et al.	2016-07-05	47
29	US-9193955-B2	Preview PDF	Markers of acute myeloid leukemia stem cells	Majeti; Ravindra et al.	2015-11-24	55
30	US-20140271683-A1	Preview PDF	Therapeutic and Diagnostic Methods for Manipulating Phagocytosis Through Calreticulin and Low Density Lipoprotein-Related Receptor	Chao; Mark P. et al.	2014-09-18	34
31	US-8758750-B2	Preview PDF	Synergistic anti-CD47 therapy for hematologic cancers	Weissman; Irving L. et al.	2014-06-24	66
32	US-20140161805-A1	Preview PDF	Methods for Manipulating Phagocytosis Mediated by CD47	Jamieson; Catriona Helen M. et al.	2014-06-12	81
33	US-20140148351-A1	Preview PDF	Prediction of Clinical Outcome in Hematological Malignancies Using a Self-Renewal Expression Signature	Alizadeh; Arash Ash et al.	2014-05-29	42
34	US-8709429-B2	Preview PDF	Markers of acute myeloid leukemia stem cells	Majeti; Ravindra et al.	2014-04-29	55
35	US-8562997-B2	Preview PDF	Methods of treating acute myeloid leukemia by blocking CD47	Jaiswal; Siddhartha et al.	2013-10-22	91
36	US-20130244326-A1	Preview PDF	Markers of Acute Myeloid Leukemia Stem Cells	Majeti; Ravindra et al.	2013-09-19	56
37	US-20130142786-A1	Preview PDF	HUMANIZED AND CHIMERIC MONOCLONAL ANTIBODIES TO CD47	Liu; Jie et al.	2013-06-06	48
38	US-8361736-B2	Preview PDF	Ex vivo methods for targeting or depleting acute myeloid leukemia cancer stem cells	Majeti; Ravindra et	2013-01-29	55
